# A bibliometric analysis of ferroptosis, necroptosis, pyroptosis, and cuproptosis in cancer from 2012 to 2022

**DOI:** 10.1038/s41420-023-01421-1

**Published:** 2023-04-15

**Authors:** Yan-Dong Miao, Wuxia Quan, Xin Dong, Jian Gan, Cui-Feng Ji, Jiang-Tao Wang, Fang Zhang

**Affiliations:** 1 The Cancer Center, Yantai Affiliated Hospital of Binzhou Medical University, The 2nd Medical College of Binzhou Medical University, Yantai, 264100 China; 2 Yantai Affiliated Hospital of Binzhou Medical University, The 2nd Medical College of Binzhou Medical University, Yantai, 264100 China; 3 Department of Gastroenterology, Yantai Affiliated Hospital of Binzhou Medical University, The 2nd Medical College of Binzhou Medical University, Yantai, 264100 China; 4 Department of Thyroid and Breast Surgery, Yantai Affiliated Hospital of Binzhou Medical University, The 2nd Medical College of Binzhou Medical University, Yantai, 264100 China

**Keywords:** Necroptosis, Tumour biomarkers

## Abstract

This study aims to visualize research hotspots and trends of “ferroptosis in cancer”, “necroptosis in cancer”, “pyroptosis in cancer”, and “cuproptosis in cancer” through a bibliometric analysis to facilitate understanding of future developments in basic and clinical research and to provide a new perspective on cancer treatment. From January 1, 2012 to October 31, 2022, in the field of “ferroptosis in cancer”, a total of 2467 organizations from 79 different countries published 3302 articles. 2274 organizations from 72 different countries published 2233 articles in the field of “ necroptosis in cancer”. 1366 institutions from 58 different countries contributed 1445 publications in the field of “pyroptosis in cancer”. In the field of “ cuproptosis in cancer”, the number of articles published in the last 10 years is relatively low, with a total of 109 articles published by 116 institutions from four different countries. In the field of “ferroptosis in cancer”, Tang Daolin had published 66 documents, ranked the first, while Dixon SJ is the most cited author, cited 3148 times; In the fields of “necroptosis in cancer”, Vandenabeele peter had published 35 papers and Degterev had been cited 995 times, ranked the first, respectively; Kanneganti thirumala-devi had published 24 papers, is the highest number of publications in the fields of “pyroptosis in cancer”, while Shi JJ was the most cited author with being cited 508 times. Both Huang Yan and Wang Tao published three papers and tied for first place and Tsvetkov p ranks first with being cited 107 times in “cuproptosis in cancer”. “Cell”, “Cell”, “Nature”, and “Science” was the most frequently co-cited journal on “ferroptosis in cancer”, “necroptosis in cancer”, “pyroptosis in cancer”, and “cuproptosis in cancer”, respectively. Further exploration of inhibitors of different Programmed cell death (PCD) and their targeted therapies are potential treatment options for cancer, but more direct clinical evidence as well as higher level clinical trials remain to be explored. Further clarification of the mechanisms of crosstalk between these PCDs may provide effective cancer treatments. And the role of different types of PCDs, especially the novel ones discovered, in cancer can be expected to remain a hot topic of research in the cancer field for quite some time to come.

## Introduction

Based on estimates from the International Agency for Research on Cancer, there will be 19.3 million new cancer cases and nearly 10 million cancer fatalities globally in 2020. Unlike statistics from previous years, female breast cancer has surpassed lung cancer as the most prevalent cancer diagnosed in 2020, with an estimated 2.3 million new cases (11.7%) [1]. According to the World Health Organization 2019 estimates, cancer is the first or second leading reason of death before age 70 in 112 of 183 countries [2]. In general, the burden of cancer incidence and mortality is increasing rapidly worldwide.

Cell death is the irreversible cessation of life phenomena. Cell death frequently occurs in normal tissues and is required to sustain tissue function and morphology, including accidental cell death (ACD) and regulated cell death (RCD), which under physiological conditions was also known as programmed cell death (PCD) [3–5]. ACD is caused by exposure to severe mechanical, physical, or chemical damage. In contrast, RCD is a form of death mediated through a signal transduction pathway and a well-defined mechanism of action [6]. RCD is divided into two categories: apoptotic and non-apoptotic [5, 6]. The common non-apoptotic PCDs are autophagy [7], ferroptosis [8], necroptosis [9], pyroptosis [10], as well as cuproptosis [11], which has been discovered in 2022. Dixon [8] found a unique iron-dependent form of non-apoptotic cell death, which is activated via the oncogenic RAS-selective lethality of the small molecule erastin, then he termed ferroptosis. Ferroptosis relies on intracellular iron, but not other metals, and is marked by oxidative damage to phospholipids [12, 13]. Necroptosis is a regulatory necrotic pathway that requires the mixed lineage kinase domain like pseudokinase (MLKL) and proteins receptor-interacting serine/threonine kinase 3 (RIPK3) and is induced via death receptors (TNF-α), interferons (IFN-α/-γ), toll-like receptors (TLR3/4/9), intracellular RNA and DNA sensors, as well as possibly other mediators [14]. Besides, MLKL and RIPK1/3were also participated in the development of necroptosis, with MLKL as the hub element [9, 15, 16]. Pyroptosis is a novel pattern of PCDs identified and proven in the latest years, which is characterized via its reliance on inflammatory cysteases (primarily caspase-1/4/5/11) and accompanied by a large release of pro-inflammatory factors [17–19]. Gasdermin D (GSDMD) activated by the inflammasome induces thermalization by the formation of membrane pores, and pyroptosis is redefined as gasdermin-mediated programmed cell necrosis [20, 21]. Caspase-1/4/5/11 specifically cleaves the junction between the carboxy-terminal gasdermin-C and amino-terminal gasdermin-N structural domains in GSDMD, which is necessary and sufficient for pyroptosis [10]. On March 17, 2022, Tsvetkov et al. reported that they found a novel mode of cell death that differs from known cell death mechanisms, the copper-dependent controlled cell death, and the team named this cell death mode - cuprotosis [11]. Cuprotosis occurs via the direct combination of copper to the lipidated components of the tricarboxylic acid (TCA) cycle. This leads to lipidated proteins accumulation and the following loss of iron-sulfur cluster proteins, causing proteotoxic stress and eventually cell death [11]. Recently, more and more evidence have indicated that ferroptosis, necroptosis, and pyroptosis act an essential role in cancer development and the association between cuproptosis and cancer occurrence has also been sporadically reported [22–25]. CD8^+^ T cells modulate tumor ferroptosis during cancer immunotherapy [26]. Zhang et al. found that the exosome miR-522 secreted via cancer-associated fibroblasts inhibited ferroptosis in gastric cancer cells via targeting ALOX15 and blocking the lipid ROS accumulation [27]. Deletion of Slc7a11 triggers tumor-selective ferroptosis and suppresses the growth of pancreatic ductal adenocarcinoma [28]. Baik et al. discovered that Z-DNA-binding protein 1 (ZBP1) expression is significantly elevated in necrotic tumors. Importantly, ZBP1 deficiency prevents tumor necrosis and inhibits metastasis during breast cancer progression [29]. In addition, MLKL might influence cancer progression and metastasis via both necroptosis-dependent and non-dependent function [30]. Gasdermin E inhibits cancer growth via activating anti-tumor immunity [31]. In addition, PD-L1-mediated gasdermin C (GSDMC) expression transforms apoptosis to pyroptosis and facilitates tumor necrosis in cancer cells [32]. miR-21-5p induces colorectal cancer pyroptosis by TGFBI modulation [33]. All this evidence indicates ferroptosis, necroptosis, pyroptosis, and cuproptosis play a crucial role in the progression and metastasis of human cancers, and all are related to the tumor microenvironment and immunotherapy of malignancies [34–36]. The novel discoveries offer potential strategies for the clinical therapy and prognosis of cancer, such as targeting ferroptosis, necroptosis, pyroptosis, and cuproptosis. However, to the best of our knowledge, there is a lack of objective and overall reporting on the general trends of publications, distribution of studies, institutions and their collaborations, and hotspot numbers in the field of “ferroptosis in cancer”, “necroptosis in cancer”, “pyroptosis in cancer”, and “cuproptosis in cancer”.

Bibliometrics is a cross-cutting science that utilizes statistical and mathematical methods to quantitatively analyze all knowledge vectors. It is an integrated body of knowledge that incorporates mathematics, statistics, and a bibliography, focusing on quantitative [37]. In this current work, we aimed to systematically discuss the study of “ferroptosis in cancer”, “necroptosis in cancer”, “pyroptosis in cancer”, and “cuproptosis in cancer” from January 1, 2012 to October 31, 2022 through a bibliometric analysis, respectively. VOSviewer software [38] was utilized to visually analyze annual publications, countries/regions, institutions, journals, authors, and co-citations; to assess global collaboration patterns among authors, institutions, and countries; and to identify research trends and hotspots in the field of “ferroptosis in cancer”, “necroptosis in cancer”, “pyroptosis in cancer”, and “cuproptosis in cancer” to supply novel ideas for basic cancer study and clinical control.

## Results

### Analysis of annual publication trends

**Fig. 1:**
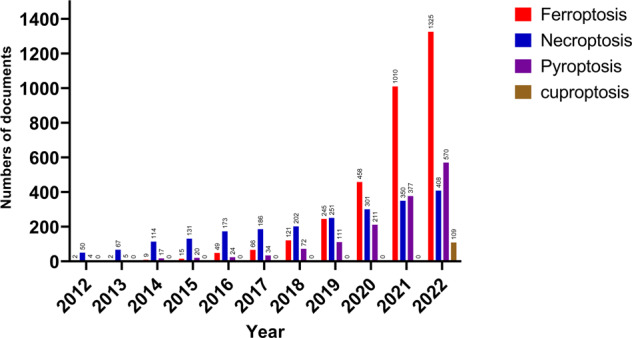
Analysis of annual publication trends. Trend of “ferroptosis in cancer”, “necroptosis in cancer”, “pyroptosis in cancer”, and “cuproptosis in cancer” publications from 2012 to 2022.

The number of publications in every period reflects the trend of study in the field. The number of articles concerning “ferroptosis in cancer”, “necroptosis in cancer”, “pyroptosis in cancer”, and “cuproptosis in cancer”, increased year by year, respectively (Fig. 1). For “ferroptosis in cancer”, publication outputs are extremely low from 2012 to 2016, and the research remained stagnant. From 2017 to 2020, the volume of literatures have steadily increased, indicating the beginning of interest in the field. From 2021 to 2022, the number of publications exploded, reaching 1325 publications as of October 31, 2022. “Necroptosis in cancer” has essentially grown steadily year by year, indicating that too much attention has been paid to this direction. Similar to “ferroptosis in cancer”, the slope of the growth trend of “pyroptosis in cancer” is greater from year to year, and the articles are growing rapidly year by year starting in 2020. Unlike “ferroptosis in cancer”, “necroptosis in cancer”, and “pyroptosis in cancer”, Cuproptosis is a novel mode of cell death discovered in 2022, and its cancer research is at the beginning stage, and overall research trend cannot be well demonstrated yet.

### Distribution of countries/ regions and organizations

In the field of “ferroptosis in cancer”, a total of 2467 organizations from 79 different countries published 3302 articles. 2274 organizations from 72 different countries published 2233 articles in the field of “ necroptosis in cancer”. 1366 institutions from 58 different countries contributed 1445 publications in the field of “pyroptosis in cancer”. In the field of “ cuproptosis in cancer”, the number of articles published in the last 10 years is relatively low, with a total of 109 articles published by 116 institutions from four different countries. Both China and the USA are in the top two countries for the number of publications on the four different types of cell death in cancer and were well ahead of other countries. In terms of total citations, the USA ranked first in “ferroptosis in cancer” and “necroptosis in cancer” with 55,450 and 33,898 times, respectively, while China ranked first in “pyroptosis in cancer” and “cuproptosis in cancer” with 17,797 and 48 times, respectively (Table 1). Among the top 10 orgnizations, except for University Ghent belongs to Belgium and University Melbourne belongs to Australia, and other institutions all belong to China. Central South University (China) ranked first in the number of publications in the field of “ferroptosis in cancer”, “pyroptosis in cancer”, and “cuproptosis in cancer”. University Ghent (Belgium) ranked first in both the number of publications and citations in the field of “necroptosis in cancer”. Guangzhou Medical University (China) ranked first in the number of citations in the field of “ferroptosis in cancer”. Chinese Academy of Science (China) ranked first in the number of citations in the field of “pyroptosis in cancer”. Central South University (China) ranked first in the number of citations in the field of “cuproptosis in cancer” (Table 1).

**Table 1:** Top 10 Countries/ Regions and orgnizations related to ferroptosis, necroptosis, pyroptosis, and cuproptosis in cancer.

Type	Rank	Country	Avg. pub. year	Documents	Citations	Rank	Orgnization	Avg. pub. year	Documents	Citations
Ferroptosis in cancer	1	Peoples R China	2021	2192	46,804	1	Central South University (China)	2021	128	1661
	2	USA	2020	629	55,450	2	Shanghai Jiao Tong University (China)	2021	112	1964
	3	Germany	2020	159	13,628	3	Zhejiang University (China)	2021	110	1822
	4	Japan	2020	157	8449	4	Chinese Academy of Science (China)	2021	101	4781
	5	France	2019	80	5407	5	Sun Yat Sen University (China)	2021	101	1779
	6	Italy	2021	79	1667	6	Guangzhou Med University (China)	2020	98	11767
	7	Australia	2020	57	4412	7	Fudan University (China)	2021	92	1473
	8	South Korea	2020	56	2448	8	Wuhan University (China)	2021	67	950
	9	Canada	2020	45	2929	9	Southern Medical University (China)	2021	66	1372
	10	Russia	2020	45	2470	10	Sichuan University (China)	2021	63	1085
Necroptosis in cancer	1	Peoples R China	2019	868	22,856	1	University Ghent (Belgium)	2018	58	5328
	2	USA	2018	539	33,898	2	Chinese Academy of Science (China)	2018	47	2922
	3	Germany	2018	235	12,094	3	Sun Yat Sen University (China)	2020	44	922
	4	South Korea	2019	137	2314	4	Central South University (China)	2021	38	342
	5	Japan	2017	97	2552	5	Shanghai Jiao Tong University (China)	2019	38	729
	6	England	2018	83	5558	6	University Melbourne (Australia)	2017	37	3165
	7	Italy	2018	78	3909	7	Zhejiang University (China)	2019	36	711
	8	Belgium	2018	72	8085	8	Fudan University (China)	2019	33	1593
	9	France	2018	69	5601	9	Sichuan University (China)	2019	32	563
	10	Australia	2018	68	6328	10	Xiamen University (China)	2018	32	2500
Pyroptosis in cancer	1	Peoples R China	2021	1012	17,797	1	Central South University (China)	2021	75	878
	2	USA	2019	222	11,027	2	Sun Yat Sen University (China)	2021	55	1103
	3	Germany	2020	43	2141	3	Nanjing Medical University (China)	2021	47	656
	4	Australia	2019	30	2569	4	Shanghai Jiao Tong University (China)	2021	47	682
	5	Japan	2019	27	675	5	Harbin Medical University (China)	2020	42	798
	6	Spain	2020	27	1262	6	Chinese Academy of Science (China)	2020	39	2511
	7	France	2019	23	1149	7	Zhejiang University (China)	2021	39	298
	8	South Korea	2020	22	344	8	Fudan University (China)	2021	32	652
	9	England	2020	19	1082	9	Wenzhou Medical University (China)	2021	32	262
	10	Iran	2021	19	354	10	Wuhan University (China)	2022	31	274
Cuproptosis in cancer	1	Peoples R China	2022	94	48	1	Central South University (China)	2022	4	4
	2	USA	2022	3	0	2	Wuhan University (China)	2022	3	0
	3	Italy	2022	1	20	3	Chengdu University Tradit Chinese Medcine (China)	2022	2	0
	4	Sweden	2022	1	0	4	Fourth Military Medical University (China)	2022	2	1
						5	Huazhong University Sci & Technol (China)	2022	2	0
						6	Jinan University (China)	2022	2	1
						7	Sun Yat Sen University (China)	2022	2	0
						8	Wenzhou Medical University (China)	2022	2	0
						9	Xi An Jiao Tong University (China)	2022	2	0
						10	Zhejiang University (China)	2022	2	19

Regardless of the co-author ship (Fig. S1), citation (Fig. S2), or bibliographic coupling analyses (Fig. S3), collaboration at the national level in the four areas of cancer research is concentrated between China and the United States, with relatively weak collaboration between other countries. Cooperation between organizations is demonstrated in co-author ship (Fig. S4), citation (Fig. S5) or bibliographic coupling analyses (Fig. S6). The top organizations show a wide range of relationships with other organizations, but some institutions are isolated.

### Co-authorship authors, citation authors, bibliographic coupling authors, and co-citation authors

A total of 16,855, 12,734, 8157, and 636 authors were contributing to the publication on “ferroptosis in cancer”, “necroptosis in cancer”, and “pyroptosis in cancer”, and “cuproptosis in cancer”, respectively. Tang Daolin had published 66 documents, ranked first in the field of “ferroptosis in cancer”, followed by Kang Rui (62 papers) and Liu Jiao (32 papers), et al. In the field of “necroptosis in cancer”, Vandenabeele peter had published the largest number of papers (35), followed by Fulda Simone (19) and Han Jiahuai (15), et al. Kanneganti Thirumala-devi had the highest number of publications (24) in the fields of “pyroptosis in cancer”, followed by Wang Wei (10) and Wang Yan (10) et al. Unlike the three research areas mentioned above, there are relatively few research articles related to “cuproptosis in cancer”, with both Huang Yan and Wang Tao published three papers and tied for first place (Table 2, Figs. S7, S8, 2). Co-citation authors are two or more authors who are simultaneously cited via another paper, and these two or more authors are composition a co-citation relationship.

**Table 2:** Top 10 authors and co-cited authors related to ferroptosis, necroptosis, pyroptosis, and cuproptosis in cancer.

Type	Rank	Author	Avg. pub. year	Documents	Citations	Rank	Co-cited author	Citations
Ferroptosis in cancer	1	Tang, Daolin	2020	66	7612	1	Dixon, SJ	3148
	2	Kang, Rui	2020	62	7569	2	Yang, WS	2492
	3	Liu, Jiao	2020	32	1702	3	Stockwell, BR	1358
	4	Stockwell, Brent R.	2018	31	13,634	4	Gao, MH	1093
	5	Conrad, Marcus	2020	30	4173	5	Doll, S	1089
	6	Toyokuni, Shinya	2020	27	532	6	Angeli, JPF	1071
	7	Gan, Boyi	2021	25	1897	7	Jiang, L	791
	8	Dixon, Scott J.	2019	22	9511	8	Hassannia, B	692
	9	Kroemer, Guido	2020	22	3171	9	Chen, X	684
	10	Chen, Xin	2021	22	1844	10	Xie, Y	653
Necroptosis in cancer	1	Vandenabeele, Peter	2018	35	3625	1	Degterev, A	995
	2	Fulda, Simone	2017	19	526	2	Galluzzi, L	957
	3	Han, Jiahuai	2017	15	2084	3	He, SD	635
	4	Linkermann, Andreas	2017	14	2167	4	Newton, K	596
	5	Yuan, Junying	2017	14	1874	5	Vandenabeele, P	569
	6	Kanneganti, Thirumala-devi	2021	13	460	6	Sun, LM	537
	7	Green, Douglas R.	2016	12	2835	7	Linkermann, A	534
	8	Kroemer, Guido	2017	12	1561	8	Cho, Y	510
	9	Balachandran, Siddharth	2017	11	328	9	Kaiser, WJ	510
	10	Efferth, Thomas	2019	11	469	10	Dondelinger, Y	504
Pyroptosis in cancer	1	Kanneganti, Thirumala-devi	2019	24	2479	1	Shi, JJ	868
	2	Wang, Wei	2021	10	59	2	Kayagaki, N	508
	3	Wang, Yan	2021	10	152	3	Wang, YP	430
	4	Karki, Rajendra	2019	9	896	4	Man, SM	426
	5	Li, Yan	2021	9	110	5	Galluzzi, L	358
	6	Man, Si Ming	2018	9	1289	6	Liu, X	339
	7	Zhang, Yan	2021	9	37	7	Broz, P	328
	8	Li, Xiaoling	2021	8	150	8	Rogers, C	310
	9	Li, Yang	2022	8	19	9	Zhang, ZB	294
	10	Liu, Yang	2021	8	70	10	Ding, JJ	287
Cuproptosis in cancer	1	Huang, Yan	2022	3	0	1	Tsvetkov, P	107
	2	Wang, Tao	2022	3	0	2	Ge, EJ	35
	3	Cai, Zhiyong	2022	2	0	3	Sung, H	35
	4	Hu, Jiao	2022	2	0	4	Siegel, RL	27
	5	Jiang, Xin	2022	2	0	5	Yoshihara, K	23
	6	Jin, Liang	2022	2	0	6	Geeleher, P	21
	7	Li, Huihuang	2022	2	0	7	Kahlson martha, A	20
	8	Li, Jianbo	2022	2	1	8	Tang, DL	20
	9	Liu, Xiang	2022	2	0	9	Jiang, P	19
	10	Liu, Yang	2022	2	4	10	Wilkerson, MD	19

**Fig. 2:**
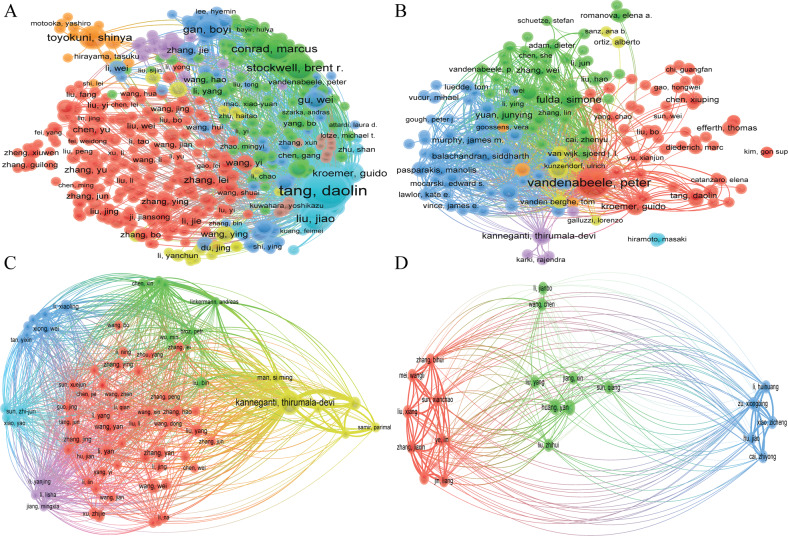
VOSviewer visualization map of Bibliographic coupling Authors. **A** ferroptosis in cancer; **B** necroptosis in cancer; **C** pyroptosis in cancer; **D** cuproptosis in cancer. Each circle indicates an author, the circle size indicates the number of citations of that author’s published articles in the bibliographic coupling analysis, the larger the circle, the higher the number of citations, the lines between the circles indicate the connections between authors, and the connection networks of different colors indicate the clusters of cooperation between different authors. Different colors represent different clusters.

**Fig. 3:**
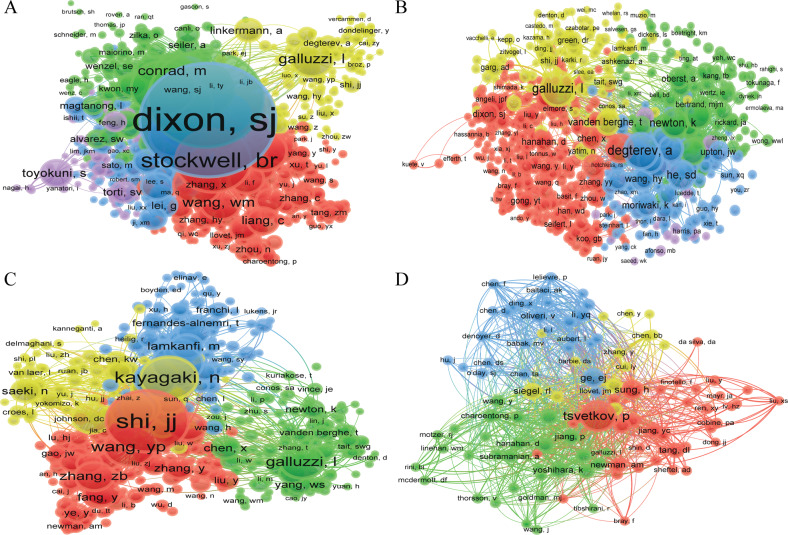
VOSviewer visualization map of co-citation authors. **A** ferroptosis in cancer; **B** necroptosis in cancer; **C** pyroptosis in cancer; **D** cuproptosis in cancer. Each circle represents an author, the circle size indicates the number of co-citations of that author’s published articles, the larger the circle, the higher the number of co-citations, the lines between the circles indicate the connections between authors, and the connection networks of different colors indicate the collaborative clusters between different authors. Different colors represent different clusters.

A total of 64,798, 57,114, 40,763, and 3245 co-citation authors were contributing to the publication of literature on “ferroptosis in cancer”, “necroptosis in cancer”, “pyroptosis in cancer”, and “cuproptosis in cancer”, respectively (Table 2). In the field of “ferroptosis in cancer”, six authors had been cited more than 1000 times, Dixon SJ was the most cited author (3148 times), followed by Yang (2492 times), et al. Degterev had been cited 995 times, ranked the first in the fields of “necroptosis in cancer”, followed by Galluzzi L (957 times) and He sd (635 times), et al. Shi JJ had been cited 508 times, ranked the first in the fields of “pyroptosis in cancer”, followed by Wang YP (430 times) and Man SM (426 times), et al. Because the role of “cuproptosis in cancer” is a very new research area, only Tsvetkov P has been cited more than 100 times so far and he ranks first with 107 times, followed by Ge EJ (35 times) and Sung H (35 times), et al. (Table 2, Fig. 3). The cooperation network between different authors in different analysis methods is shown in Figs. S7, S8, and 2, respectively.

### Citation journal, bibliographic journal, and co-citation journal

**Fig. 4:**
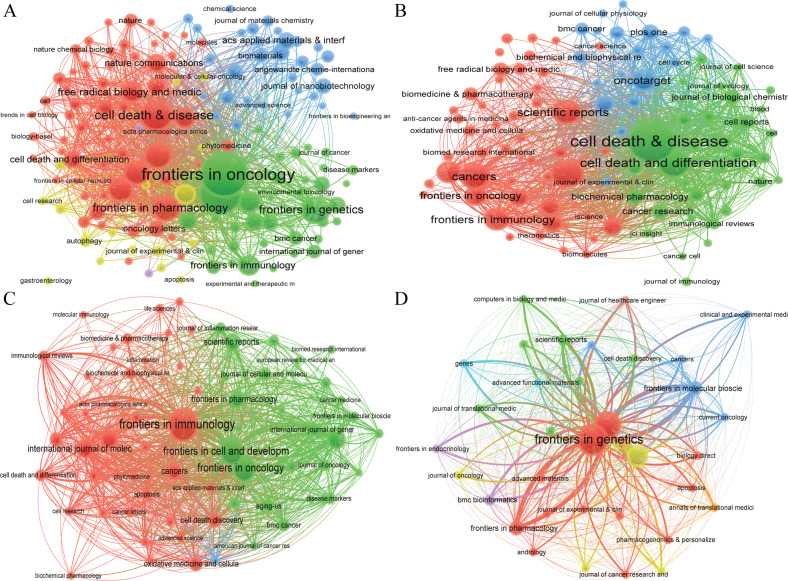
VOSviewer visualization map of bibliographic coupling source. **A** ferroptosis in cancer; **B** necroptosis in cancer; **C** pyroptosis in cancer; **D** cuproptosis in cancer. Each circle indicates a journal, the circle size indicates the number of publications in that journal in the bibliographic coupling analysis, the larger the circle, the higher the number of publications, the lines between the circles indicate the connections between journals, and the connection networks of different colors indicate the clusters of cooperation between different journals. Different colors represent different clusters.

We found that a total of 3236 articles were published on 697 academic journals in the field of “ferroptosis in cancer”. 695 academic journals published 2203 papers in the field of “necroptosis in cancer”. 497 academic journals published 1427 papers in the field of “pyroptosis in cancer”. 95 articles were published on 34 academic journals in the field of “cuproptosis in cancer”. The journal “Frontiers in oncology” (137 articles) had the highest number of outputs in the fields of “ferroptosis in cancer”, followed by “Frontiers in cell and developmental biology” (113 articles) and “Cell death & disease” (76 articles), et al. In the fields of “necroptosis in cancer”, the journal “Cell death & disease” (98 articles) had the highest number of outputs, followed by “Cell death and differentiation” (55 articles) and “International journal of molecular sciences” (53 articles), et al. The journal “Frontiers in immunology” had published 67 articles, ranked first in the fields of “pyroptosis in cancer”, followed by “Frontiers in cell and developmental biology” (48 articles) and “Frontiers in oncology” (47 articles), et al. In the fields of “cuproptosis in cancer”, the journal “Frontiers in genetics” (26 articles) had the highest number output, followed by “Frontiers in immunology” (19 articles) and “Frontiers in oncology” (11 articles), et al. Among the top 10 academic journals, the highest impact factor (IF) was “Cell death & disease” (IF:9.685) in the field of “ferroptosis in cancer”, “Cell death and differentiation” (IF:12.067) in the field of “necroptosis in cancer”, “ Cell death & disease” (IF:9.685) in the field of “pyroptosis in cancer”, and “Advanced functional materials” (IF:19.924) in the field of “cuproptosis in cancer”, respectively. Besides, more than 50% of the top 10 journals belong to Journal Citation Reports (JCR) Quartile in Category 1 (Q1) (Table 3, Figs. 4, S9).

**Table 3:** Top 10 journals and co-cited journals related to ferroptosis, necroptosis, pyroptosis, and cuproptosis in cancer.

Type	Rank	Journal	Documents	Citations	IF (2021)	JCR	Rank	Co-cited journal	Citations	IF (2021)	JCR
Ferroptosis in cancer	1	Frontiers in oncology	137	917	5.738	Q2	1	Cell	8158	66.85	Q1
	2	Frontiers in cell and developmental biology	113	863	6.081	Q1	2	Nature	7551	69.504	Q1
	3	Cell death & disease	76	2491	9.685	Q1	3	Proc Natl Acad Sci USA	4295	12.779	Q1
	4	Frontiers in genetics	62	184	4.772	Q2	4	J Biol Chem	3747	5.486	Q2
	5	Oxidative medicine and cellular longevity	59	843	7.310	Q2	5	Cell Death Differ	3555	12.067	Q1
	6	Frontiers in pharmacology	58	697	5.988	Q1	6	Cancer Res	3366	13.312	Q1
	7	Biochemical and biophysical research communications	55	2059	3.322	Q2	7	Free Radical Bio Med	3099	8.101	Q1
	8	International journal of molecular sciences	52	883	6.208	Q1	8	Cell Death Dis	2889	9.685	Q1
	9	Free radical biology and medicine	43	2009	8.101	Q1	9	Nat Commun	2814	17.694	Q1
	10	Frontiers in molecular biosciences	43	112	6.113	Q2	10	Nat Chem Biol	2563	16.174	Q1
Necroptosis in cancer	1	Cell death & disease	98	4263	9.685	Q1	1	Cell	6520	66.85	Q1
	2	Cell death and differentiation	55	8423	12.067	Q1	2	Nature	5935	69.504	Q1
	3	International journal of molecular sciences	53	654	6.208	Q1	3	Cell Death Differ	4763	12.067	Q1
	4	Cancers	47	550	6.575	Q1	4	J Biol Chem	4146	5.486	Q2
	5	Scientific reports	43	660	4.996	Q1	5	Proc Natl Acad Sci USA	3937	12.779	Q1
	6	Frontiers in immunology	37	303	8.786	Q1	6	Cell Death Dis	2875	9.685	Q1
	7	Oncotarget	36	1426			7	Science	2758	63.714	Q1
	8	Frontiers in genetics	35	41	4.772	Q2	8	Mol Cell	2742	19.328	Q1
	9	Frontiers in oncology	31	102	5.738	Q2	9	Immunity	2133	43.474	Q1
	10	Cells	26	351	7.666	Q2	10	Cancer Res	2107	13.312	Q1
Pyroptosis in cancer	1	Frontiers in immunology	67	864	8.786	Q1	1	Nature	5520	69.504	Q1
	2	Frontiers in cell and developmental biology	48	313	6.081	Q1	2	Cell	2808	66.85	Q1
	3	Frontiers in oncology	47	397	5.738	Q2	3	Proc Natl Acad Sci USA	2369	12.779	Q1
	4	Frontiers in genetics	31	58	4.772	Q2	4	Science	1805	63.714	Q1
	5	International journal of molecular sciences	28	510	6.208	Q1	5	J Biol Chem	1733	5.486	Q2
	6	Cell death & disease	27	1296	9.685	Q1	6	J Immunol	1715	5.426	Q2
	7	Frontiers in pharmacology	23	96	5.988	Q1	7	Cell Death Differ	1712	12.067	Q1
	8	Scientific reports	22	424	4.996	Q1	8	Cell Death Dis	1698	9.685	Q1
	9	Cancers	21	258	6.575	Q1	9	Immunity	1568	43.474	Q1
	10	Cell death discovery	17	433	7.109	Q2	10	Nat Commun	1520	17.694	Q1
Cuproptosis in cancer	1	Frontiers in genetics	26	6	4.772	Q2	1	Science	150	63.714	Q1
	2	Frontiers in immunology	19	5	8.786	Q1	2	Cancer Res	91	13.312	Q1
	3	Frontiers in oncology	11	2	5.738	Q2	3	Nature	89	69.504	Q1
	4	Frontiers in molecular biosciences	4	20	6.113	Q2	4	Nat Commun	85	17.694	Q1
	5	Frontiers in pharmacology	4	6	5.988	Q1	5	CA-Cancer J Clin	79	286.13	Q1
	6	Scientific reports	2	5	4.996	Q1	6	Front Immunol	76	8.786	Q1
	7	Advanced functional materials	1	0	19.924	Q1	7	Nat Rev Cancer	74	69.8	Q1
	8	Annals of translational medicine	1	0	3.616	Q3	8	Cell	73	66.85	Q1
	9	Cancers	1	0	6.575	Q1	9	Front Oncol	62	5.738	Q2
	10	Clinical and experimental medicine	1	0	5.057	Q3	10	J Clin Oncol	62	50.717	Q1

**Fig. 5:**
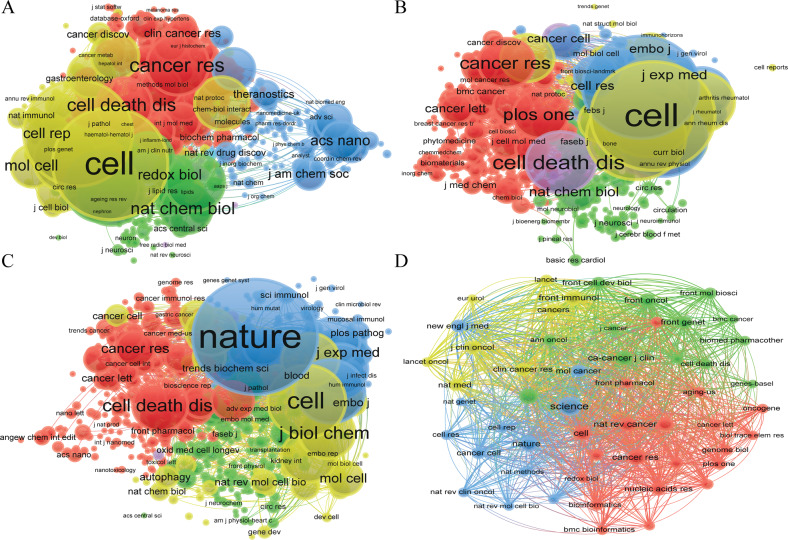
VOSviewer visualization map of co-citation source. **A** ferroptosis in cancer; **B** necroptosis in cancer; **C** pyroptosis in cancer; **D** cuproptosis in cancer. Each circle represents an journal, the circle size indicates the number of co-citations in that journal, the larger the circle, the higher the number of co-citations, the lines between the circles indicate the connections between journals, and the connection networks of different colors indicate the collaborative clusters between different journals. Different colors represent different clusters.

Co-citation journal analysis indicated that the top two most cited journals are “Cell” and “Nature” in the fields of “ferroptosis in cancer” and “necroptosis in cancer”, respectively, both with more than 5000 citations. Slightly different from the two fields mentioned above, in the field of “pyroptosis in cancer”, “Nature” is the most frequently cited journal, followed by “Cell”. The journals with the highest number of citations are “Science” (150 times) in the field of “cuproptosis in cancer”, followed by “Cancer research” (91) (Table 3, Fig. 5).

### Citation and co-cited references

**Fig. 6:**
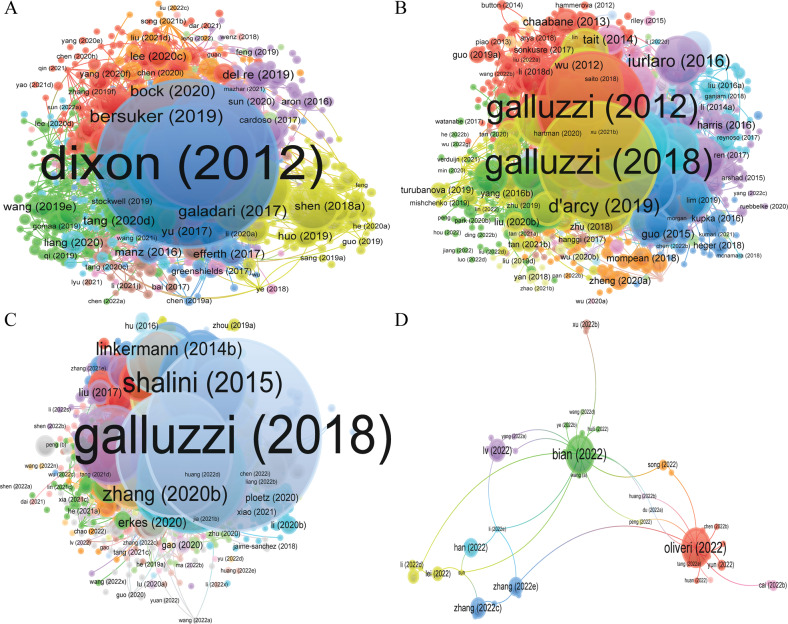
VOSviewer visualization map of citation documents. **A** ferroptosis in cancer; **B** necroptosis in cancer; **C** pyroptosis in cancer; **D** cuproptosis in cancer. Each circle indicates a document, the size of the circle indicates the number of citations, the larger the circle, the higher the number of citations, the lines between the circles indicate the connection between documents, and the connection networks of different colors indicate the clusters of cooperation between different documents. Different colors represent different clusters.

**Fig. 7:**
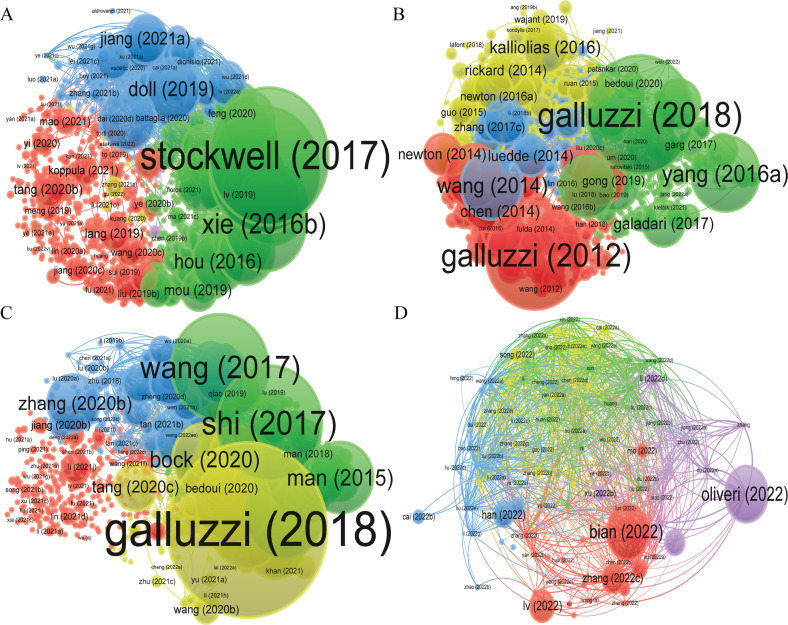
VOSviewer visualization map of bibliographic coupling documents. **A** ferroptosis in cancer; **B** necroptosis in cancer; **C** pyroptosis in cancer; **D** cuproptosis in cancer. Each circle indicates a document, the circle size indicates the number of citations in the bibliographic coupling analysis, the larger the circle, the higher the number of citations, the lines between the circles indicate the connections between documents, and the connection networks of different colors indicate the clusters of cooperation between different documents. Different colors represent different clusters.

Table 4 indicated the top 10 highly cited references of documents on “ferroptosis in cancer”, “necroptosis in cancer”, “pyroptosis in cancer”, and “cuproptosis in cancer”. “ferroptosis: an iron-dependent form of nonapoptotic cell death” [8] is the most cited reference (4558 times) in “ferroptosis in cancer”. The “molecular mechanisms of cell death: recommendations of the nomenclature committee on cell death 2018” had the highest number of citation in the fields of “necroptosis in cancer” (1995 times) and had been cited 1087 times, ranked the first in the fields of “pyroptosis in cancer”. Unlike the three research areas mentioned above, there are relatively few research documents related to “cuproptosis in cancer”, “selective targeting of cancer cells by copper ionophores: an overview” had the highest number of citations (20 times). (Table 4, Figs. 6, 7).

**Table 4:** Top 10 references related to ferroptosis, necroptosis, pyroptosis, and cuproptosis in cancer.

Type	Rank	Literature	Title	DOI	Source	IF/JCR	Citations	Ref
Ferroptosis in cancer	1	Dixon (2012)	Ferroptosis: an iron-dependent form of nonapoptotic cell death	10.1016/j.cell.2012.03.042	Cell	66.85/Q1	4558	[8]
	2	Yang (2014)	Regulation of ferroptotic cancer cell death by GPX4	10.1016/j.cell.2013.12.010	Cell	66.85/Q1	2301	[41]
	3	Stockwell (2017)	Ferroptosis: A Regulated Cell Death Nexus Linking Metabolism, Redox Biology, and Disease	10.1016/j.cell.2017.09.021	Cell	66.85/Q1	2258	[22]
	4	Galluzzi (2018)	Molecular mechanisms of cell death: recommendations of the Nomenclature Committee on Cell Death 2018	10.1038/s41418-017-0012-4	Cell death and differentiation	12.067/Q1	1995	[3]
	5	Xie (2016b)	Ferroptosis: process and function	10.1038/cdd.2015.158	Cell death and differentiation	12.067/Q1	1296	[117]
	6	Angeli (2014)	Inactivation of the ferroptosis regulator Gpx4 triggers acute renal failure in mice	10.1038/ncb3064	Nature Cell Biology	28.231/Q1	1294	[118]
	7	Jiang (2015a)	Ferroptosis as a p53-mediated activity during tumor suppression	10.1038/nature14344	Nature	69.504/Q1	1149	[46]
	8	Dixon (2014b)	The role of iron and reactive oxygen species in cell death	10.1038/nchembio.1416	Nature Chemical Biology	16.174/Q1	1130	[119]
	9	Yang (2016)	Ferroptosis: Death by Lipid Peroxidation	10.1016/j.tcb.2015.10.014	Trends in cell biology	21.167/Q1	1051	[120]
	10	Doll (2017)	ACSL4 dictates ferroptosis sensitivity by shaping cellular lipid composition	10.1038/nchembio.2239	Nature Chemical Biology	16.174/Q1	1037	[39]
Necroptosis in cancer	1	Galluzzi (2018)	Molecular mechanisms of cell death: recommendations of the Nomenclature Committee on Cell Death 2018	10.1038/s41418-017-0012-4	Cell death and differentiation	12.067/Q1	1995	[3]
	2	Galluzzi (2012)	Molecular definitions of cell death subroutines: recommendations of the Nomenclature Committee on Cell Death 2012	10.1038/cdd.2011.96	Cell death and differentiation	12.067/Q1	1743	[121]
	3	Yang (2016a)	Ferroptosis: Death by Lipid Peroxidation	10.1016/j.tcb.2015.10.014	Trends in cell biology	21.167/Q1	1051	[120]
	4	Wang (2014)	Mixed lineage kinase domain-like protein MLKL causes necrotic membrane disruption upon phosphorylation by RIP3	10.1016/j.molcel.2014.03.003	Molecular cell	19.328/Q1	906	[122]
	5	Kaczmarek (2013)	Necroptosis: the release of damage-associated molecular patterns and its physiological relevance	10.1016/j.immuni.2013.02.003	Immunity	43.474/Q1	820	[123]
	6	Gao (2015)	Glutaminolysis and Transferrin Regulate Ferroptosis	10.1016/j.molcel.2015.06.011	Molecular cell	19.328/Q1	783	[124]
	7	Cai (2014)	Plasma membrane translocation of trimerized MLKL protein is required for TNF-induced necroptosis	10.1038/ncb2883	Nature Cell Biology	28.231/Q1	773	[125]
	8	Shalini (2015)	Old, new and emerging functions of caspases	10.1038/cdd.2014.216	Cell death and differentiation	12.067/Q1	721	[126]
	9	Linkermann (2014a)	Necroptosis	10.1056/nejmra1310050			708	
	10	Murphy (2013)	The pseudokinase MLKL mediates necroptosis via a molecular switch mechanism	10.1016/j.immuni.2013.06.018	Immunity	43.474/Q1	699	[127]
Pyroptosis in cancer	1	Galluzzi (2018)	Molecular mechanisms of cell death: recommendations of the Nomenclature Committee on Cell Death 2018	10.1038/s41418-017-0012-4	Cell death and differentiation	12.067/Q1	1995	[3]
	2	Shi (2017)	Pyroptosis: Gasdermin-Mediated Programmed Necrotic Cell Death	10.1016/j.tibs.2016.10.004	Trend in biochemical sciences	14.264/Q1	1087	[21]
	3	Wang (2017)	Chemotherapy drugs induce pyroptosis through caspase-3 cleavage of a gasdermin	10.1038/nature22393	Nature	69.504/Q1	979	[89]
	4	Shalini (2015)	Old, new and emerging functions of caspases	10.1038/cdd.2014.216	Cell death and differentiation	12.067/Q1	721	[126]
	5	D’arcy (2019)	Cell death: a review of the major forms of apoptosis, necrosis and autophagy	10.1002/cbin.11137	Cell bilology international	4.473/Q4	685	[128]
	6	Tang (2019)	The molecular machinery of regulated cell death	10.1038/s41422-019-0164-5	Cell research	46.297/Q1	655	[6]
	7	Bock (2020)	Mitochondria as multifaceted regulators of cell death	10.1038/s41580-019-0173-8	Nature Reviews molecular cell biology	113.915/Q1	564	[129]
	8	Man (2015)	Regulation of inflammasome activation	10.1111/imr.12296	Immunological Reviews	10.983/Q1	550	[130]
	9	Chen (2014)	Translocation of mixed lineage kinase domain-like protein to plasma membrane leads to necrotic cell death	10.1038/cr.2013.171	Cell research	46.297/Q1	489	[131]
	10	Zhang (2018c)	Plasma membrane changes during programmed cell deaths	10.1038/cr.2017.133	Cell research	46.297/Q1	414	[132]
Cuproptosis in cancer	1	Oliveri (2022)	Selective Targeting of Cancer Cells by Copper Ionophores: An Overview	10.3389/fmolb.2022.841814	Frontiers in molecular biosciences	6.113/Q3	20	[40]
	2	Bian (2022)	A Novel Cuproptosis-Related Prognostic Gene Signature and Validation of Differential Expression in Clear Cell Renal Cell Carcinoma	10.3390/genes13050851	Genes(Basel)	4.141/Q3	19	[133]
	3	Lv (2022)	Comprehensive Analysis of Cuproptosis-Related Genes in Immune Infiltration and Prognosis in Melanoma	10.3389/fphar.2022.930041	Frontiers in pharmacology	5.988/Q2	6	[134]
	4	Zhang (2022c)	A novel Cuproptosis-related LncRNA signature to predict prognosis in hepatocellular carcinoma	10.1038/s41598-022-15251-1	Scientific reports	4.996/Q3	5	[135]
	5	Han (2022)	A Newly Established Cuproptosis-Associated Long Non-Coding RNA Signature for Predicting Prognosis and Indicating Immune Microenvironment Features in Soft Tissue Sarcoma	10.1155/2022/8489387	Journal of oncology	4.501/Q4	4	[136]
	6	Zhang (2022e)	Cuproptosis-Related Risk Score Predicts Prognosis and Characterizes the Tumor Microenvironment in Hepatocellular Carcinoma	10.3389/fimmu.2022.925618	Frontiers in immunology	8.786/Q2	4	[137]
	7	Lei (2022)	A novel cuproptosis-related gene signature for predicting prognosis in cervical cancer	10.3389/fgene.2022.957744	Frontiers in genetics	4.772/Q3	2	[138]
	8	Yang (2022b)	Cuproptosis-Related lncRNAs are Biomarkers of Prognosis and Immune Microenvironment in Head and Neck Squamous Cell Carcinoma	10.3389/fgene.2022.947551	Frontiers in genetics	4.772/Q3	2	[139]
	9	Song (2022)	Cuproptosis scoring system to predict the clinical outcome and immune response in bladder cancer	10.3389/fimmu.2022.958368	Frontiers in immunology	8.786/Q2	1	[23]
	10	Xu (2022b)	Cuproptosis-Associated lncRNA Establishes New Prognostic Profile and Predicts Immunotherapy Response in Clear Cell Renal Cell Carcinoma	10.3389/fgene.2022.938259	Frontiers in genetics	4.772/Q3	1	[140]

**Fig. 8:**
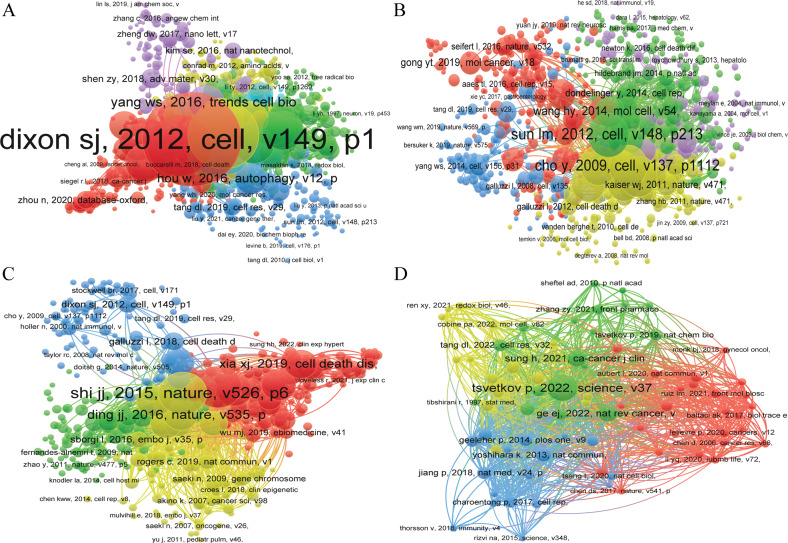
VOSviewer visualization map of co-citation references. **A** Ferroptosis in cancer; **B** necroptosis in cancer; **C** pyroptosis in cancer; **D** cuproptosis in cancer. Each circle represents an reference, the circle size indicates the number of reference co-citations, the larger the circle, the higher the number of co-citations, the lines between the circles indicate the connections between references, and the connection networks of different colors indicate the collaborative clusters between different references. Different colors represent different clusters.

Table 5 and Fig. 8 showed the top 10 highly co-cited references on “ferroptosis in cancer”, “necroptosis in cancer”, “pyroptosis in cancer”, and “cuproptosis in cancer”. Table S1 and Fig. S10 showed the keyword of the co-occurrence subnetwork. The top 10 keywords in terms of occurrence were “ferroptosis”, “prognosis”, “apoptosis”, “cancer”, “iron”, “lipid peroxidation”, “autophagy”, “cell death”, “Gpx4”, and “immunotherapy” in the field of “ferroptosis in cancer”. “Necroptosis”, “apoptosis”, “autophagy”, “cell death”, “cancer”, “ferroptosis”, “inflammation”, “pyroptosis”, “necrosis”, and “Mlkl” are located in the top 10 keywords in terms of occurrence in the field of “necroptosis in cancer”. In the field of “pyroptosis in cancer”, “pyroptosis”, “apoptosis”, “prognosis”, “inflammasome”, “immunotherapy”, “necroptosis”, “inflammation”, “ferroptosis”, “autophagy”, and “tumor microenvironment” were the top 10 keywords in terms of occurrence. “Cuproptosis”, “prognosis”, “immunotherapy”, “tumor microenvironment”, “lncRNA”, “prognostic signature”, “lung adenocarcinoma”, “overall survival”, “prognostic model”, and “drug sensitivity” located in the top five keywords in terms of occurrence in the field of “cuproptosis in cancer”.

**Table 5:** Top 10 co-cited references associated with ferroptosis, necroptosis, pyroptosis, and cuproptosis in cancer.

Type	Rank	Reference	Co-citations	Year
Ferroptosis in cancer	1	Dixon SJ, 2012, cell, v149, p1060, 10.1016/j.cell.2012.03.042	1991	2012
	2	Yang WS, 2014, cell, v156, p317, 10.1016/j.cell.2013.12.010	1244	2014
	3	Stockwell BR, 2017, cell, v171, p273, 10.1016/j.cell.2017.09.021	1077	2017
	4	Jiang L, 2015, nature, v520, p57, 10.1038/nature14344	677	2015
	5	Xie Y, 2016, cell death differ, v23, p369, 10.1038/cdd.2015.158	625	2016
	6	Angeli JPF, 2014, nat cell biol, v16, p1180, 10.1038/ncb3064	574	2014
	7	Doll S, 2017, nat chem biol, v13, p91, [10.1038/nchembio.223910.1038/nchembio.2239]	550	2017
	8	Wang WM, 2019, nature, v569, p270, 10.1038/s41586-019-1170-y	545	2019
	9	Hassannia B, 2019, cancer cell, v35, p830, 10.1016/j.ccell.2019.04.002	523	2019
	10	Dixon SJ, 2014, elife, v3, 10.7554/elife.02523	486	2014
Necroptosis in cancer	1	Sun LM, 2012, cell, v148, p213, 10.1016/j.cell.2011.11.031	518	2012
	2	Cho Y, 2009, cell, v137, p1112, 10.1016/j.cell.2009.05.037	478	2009
	3	He SD, 2009, cell, v137, p1100, 10.1016/j.cell.2009.05.021	465	2009
	4	Degterev A, 2005, nat chem biol, v1, p112, 10.1038/nchembio711	451	2005
	5	Vandenabeele P, 2010, nat rev mol cell bio, v11, p700, 10.1038/nrm2970	411	2010
	6	Zhang DW, 2009, science, v325, p332, 10.1126/science.1172308	392	2009
	7	Degterev A, 2008, nat chem biol, v4, p313, 10.1038/nchembio.83	383	2008
	8	Wang HY, 2014, mol cell, v54, p133, 10.1016/j.molcel.2014.03.003	317	2014
	9	Pasparakis M, 2015, nature, v517, p311, 10.1038/nature14191	300	2015
	10	Cai ZY, 2014, nat cell biol, v16, p55, 10.1038/ncb2883	283	2014
Pyroptosis in cancer	1	Shi JJ, 2015, nature, v526, p660, 10.1038/nature15514	440	2015
	2	Wang YP, 2017, nature, v547, p99, 10.1038/nature22393	421	2017
	3	Shi JJ, 2017, trends biochem sci, v42, p245, 10.1016/j.tibs.2016.10.004	284	2017
	4	Kayagaki N, 2015, nature, v526, p666, 10.1038/nature15541	277	2015
	5	Zhang ZB, 2020, nature, v579, p415, 10.1038/s41586-020-2071-9	268	2020
	6	Ding JJ, 2016, nature, v535, p111, 10.1038/nature18590	266	2016
	7	Xia XJ, 2019, cell death dis, v10, 10.1038/s41419-019-1883-8	245	2019
	8	Liu X, 2016, nature, v535, p153, 10.1038/nature18629	241	2016
	9	Bergsbaken T, 2009, nat rev microbiol, v7, p99, 10.1038/nrmicro2070	222	2009
	10	Rogers C, 2017, nat commun, v8, 10.1038/ncomms14128	209	2017
Cuproptosis in cancer	1	Tsvetkov P, 2022, science, v375, p1254, 10.1126/science.abf0529	90	2022
	2	Ge EJ, 2022, nat rev cancer, v22, p102, 10.1038/s41568-021-00417-2	35	2022
	3	Sung H, 2021, ca-cancer j clin, v71, p209, 10.3322/caac.21660	35	2021
	4	Yoshihara K, 2013, nat commun, v4, 10.1038/ncomms3612	23	2013
	5	Kahlson Martha A, 2022, science, v375, p1231, 10.1126/science.abo3959	20	2022
	6	Geeleher P, 2014, plos one, v9, 10.1371/journal.pone.0107468	19	2014
	7	Jiang P, 2018, nat med, v24, p1550, 10.1038/s41591-018-0136-1	19	2018
	8	Wilkerson MD, 2010, bioinformatics, v26, p1572, 10.1093/bioinformatics/btq170	19	2010
	9	Hanzelmann S, 2013, bmc bioinformatics, v14, 10.1186/1471-2105-14-7	18	2013
	10	Tang DL, 2022, cell res, v32, p417, 10.1038/s41422-022-00653-7	18	2022

## Discussion

### General overview

PCD is a hot topic in the field of biological and medical studies. Research on “ferroptosis in cancer”, “necroptosis in cancer”, and “pyroptosis in cancer” had developed rapidly over the past few years and has been the focus of scholars and organizations. The number and trend of publications per year indicate the development rate and study progress of this research, as well as the concentration of study in this field. Since ferroptosis was defined as a new category of PCD by Dixon et al. [8] in 2012, it has quickly become a focus of attention and discussion due to its important function in vivo, and publications on the subject have increased year by year, especially in the field of cancer research. In particular, publication outputs are extremely low from 2012 to 2016, suggesting that research in this period was in its infancy. From 2017 to 2020, the volume of literature has steadily enhanced, indicating the beginning of interest in the field of “ferroptosis in cancer”. During this period, many heavyweight studies continue to emerge, further driving the research fervor for “ferroptosis in cancer”. Such as Stockwell et al. further detailed a summary of the basic mechanisms of ferroptosis, emphasizing the links with other fields of biology and medicine [22]. ACSL4 determines susceptibility to ferroptosis by shaping cellular lipid composition [39]. From 2021 to 2022, the number of publications exploded, reaching 1325 publications as of October 31, 2022. Thus, it can be seen that the research associated with “ferroptosis in cancer” is a hot research area in recent years, and this hotness will continue with good development trends in the future. “Necroptosis in cancer” has essentially grown steadily year by year, indicating that too much attention has been paid to this direction. Similar to “ferroptosis in cancer”, the slope of the growth trend of “pyroptosis in cancer” is greater from year to year, and the articles on “pyroptosis in cancer” are growing rapidly year by year starting in 2020. In 2015, Shi et al. [10] proposed that “GSDMD cleavage by inflammatory cystathionases determines pyroptosis cell death”, which greatly promoted the research of “pyroptosis in cancer”, followed by the rapid development of this field, and it is expected that there is still a good momentum in the future. Unlike “ferroptosis in cancer”, “necroptosis in cancer”, and “pyroptosis in cancer”, “cuproptosis” is a novel mode of cell death discovered in 2022, and its research in cancer is at the beginning stage, and overall research trend cannot be well demonstrated yet. But on the other hand, in the field of “cuproptosis in cancer” still has a large number of research directions that deserve more in-depth study in the future.

Based on the distribution of countries/regions and organizations in Table 1, China and USA both rank in the top two in terms of the number of publications in the four different RCDs in cancer, far ahead of other countries/regions. This demonstrates that China and the USA are leading the way in the above-mentioned research areas. Among the top 10 organizations, except for University Ghent belongs to Belgium and University, Melbourne belongs to Australia, and other institutions all belong to China. It can be seen that at present, Chinese institutions are at the international leading stage in terms of both the number of publications and the total number of citations. However, as we can see in Figs. S1–S6, the distribution of individual countries and organizations is dispersed, as well as the primary research targets (e.g., China and the USA, the Central South University, Shanghai Jiao Tong University, Guangzhou Medical University, University Ghent, and Chinese Academy of Science) do not form a clear network, and there is even no connection between some research organizations and others, suggesting a lack of academic communication between these countries and research organizations. The emergence of this situation can hinder the development of related study areas. Therefore, it is highly advised that academic barriers should be removed and collaboration and communication should take place between all research institutions to facilitate the rapid development in the field of “ferroptosis in cancer”, “necroptosis in cancer”, and “pyroptosis in cancer”.

From the viewpoint of authors and co-cited authors (Table 2, Figs. S2–S5), Tang daolin had published 66 documents, ranked first in the fields of “ferroptosis in cancer”, followed by Kang rui (62 documents) and Liu jiao (32 documents), et al. However, although Stockwell Brent R. and Dixon Scott J. published fewer articles than the top three authors, the number of citations of their articles was significantly higher than the former (13434, 9511 Vs 7612, 7569, 1702), indicating the higher quality of their papers and further indicating that Stockwell Brent R. and Dixon Scott J. are the core leaders in the fields of “ferroptosis in cancer”. The co-citation authors analysis further validates our view that both of them are in the top three co-cited authors. In the field of “necroptosis in cancer”, Vandenabeele peter published the largest number of papers (35 articles), followed by Fulda Simone (19 articles) and Han Jiahuai (15 articles), et al. There are relatively many representative scholars in this research area, mainly focusing on Vandenabeele Peter; Fulda Simone; Green Douglas R; Han Jiahuai; Degterev A; and Galluzzi L, et al. Kanneganti Thirumala-devi had the highest number of publications (24 articles) in the fields of “pyroptosis in cancer”, followed by Wang Wei (10 articles) and Wang Yan (10 articles) et al. The core scholars in the field of “pyroptosis in cancer” are between the study area of “ferroptosis in cancer” and “necroptosis in cancer”, mainly including Kanneganti Thirumala-devi, Man Si Ming, Shi JJ, Karki Rajendra, Kayagaki N, and so on. It shows that they are leading the research direction. Unlike the three research areas mentioned above, there are relatively few research articles related to “cuproptosis in cancer”, with both Huang Yan and Wang Tao publishing three papers and tying for first place. Tsvetkov P is the undisputed leader in this field of research, having first reported that copper induces cell death by targeting lipid acylated tricarboxylic acid (TCA) cycle [11].

In the journals and co-cited journals analysis, we found that more than 50% of the top 10 journals belong to Q1 (Table 3, Figs. S9, 4). Co-citation journal analysis indicated that the top two most cited journals are “Cell” and “Nature” in the fields of “ferroptosis in cancer” and “necroptosis in cancer”, respectively, both with more than 5000 citations. Slightly different from the two fields mentioned above, in the field of “pyroptosis in cancer”, “Nature” is the most frequently cited journal, followed by “Cell”. The journal with the highest number of citations is “Science” (150) in the field of “cuproptosis in cancer”, followed by “Cancer research” (91) (Table 3, Fig. 5). In summary, the research areas of “ferroptosis in cancer”, “necroptosis in cancer”, “pyroptosis in cancer”, and “cuproptosis in cancer” are a current hot spot and has a good trend in the future. Meanwhile, the cited literature is from high-impact top journals, which indicates that the above four research areas are also highly regarded areas in the global academic community.

Among the top 10 most cited literatures, “ferroptosis: an iron-dependent form of nonapoptotic cell death” [8] was the most cited reference (4558 times) in “ferroptosis in cancer”. “Molecular mechanisms of cell death: recommendations of the nomenclature committee on cell death 2018 [3]” had the highest number of citations (1995 times) in the fields of “necroptosis in cancer” and had been cited 1087 times, ranked the first in the fields of “pyroptosis in cancer”. “selective targeting of cancer cells by copper ionophores: an overview [40]” had the highest number of citations (20 times) in the field of “cuproptosis in cancer” (Table 4, Figs. 6, 7). Except for “cuproptosis in cancer”, where the quality of articles is relatively low, the top 10 articles in terms of citations in the field of “ferroptosis in cancer”, “necroptosis in cancer”, and “pyroptosis in cancer” are of high quality and are all published in JCR Q1 journals. Thus, these three research areas are still at the frontier of cancer research and are still hot research directions that deserve further in-depth exploration in the future. In contrast, “cuproptosis in cancer” has only just begun and is in its infancy, and there is great room for future research.

### Research hotspots, frontiers, and prospects

As the research topic and core element of the literature, keywords can reflect the distribution and development of diverse study hot spots in a specific field. Table S1 and Fig. S10 showed the keyword of the Co-occurrence subnetwork. Through this network we can clarify the study hotspots and development frontiers of “ferroptosis in cancer”, “necroptosis in cancer”, “pyroptosis in cancer”, and “cuproptosis in cancer”, which are summarized as follows:

### Ferroptosis in cancer

2012 Dixon et al. [8]. Found a new form of cell death, an iron-dependent form of PCD, named ferroptosis. The main mechanism is the induction of cell death via facilitating lipid peroxidation of unsaturated fatty acids highly expressed on cell membranes in the presence of divalent iron or ester oxygenase; in addition, it is manifested by a decrease in the regulatory core enzyme glutathione peroxidase 4 (GPX4) of the glutathione (GSH) system [41]. GPX4 utilized GSH as a reducing cofactor that decreases hydroperoxide derivatives of lipids (PLOOH) to fatty alcohol, thereby suppressing ferroptosis in cancer cells [42–44]. The ferroptosis suppressor protein 1 (FSP1)- coenzyme Q10 (CoQ10)-Nicotinamide Adenine Dinucleotide Phosphate (NADPH) pathway exists as an independent parallel system that acts in concert with GPX4 and glutathione to suppress phospholipid peroxidation and ferroptosis [13, 45]. Ferroptosis is associated with the anti-tumor function of several tumor suppressors, such as BRCA1-associated protein 1 (BAP1), p53, et al. SLC7A11 is highly expressed in human cancers, and its over-expression suppresses ROS-induced ferroptosis and eliminates p53(3KR)-mediated tumor growth inhibition in xenograft models [46]. Jennis et al. [47] reported that a prevalent single nucleotide polymorphism (SNP) in p53 detected in individuals of African descent is related to increased cancer incidence in mouse models, and cells carrying this mutation resist ferroptosis through modifying glutamine metabolism. BAP1 inhibits tumor development, in part, via SLC7A11 and ferroptosis [48]. A study reported that the p62-Keap1-NRF2 pathway activation prevented ferroptosis in hepatocellular carcinoma (HCC) cells [49]. Another study showed that intercellular interactions determine iron death in cancer cells through NF2-YAP signaling [50]. As demonstrated in the results of the co-occurrence analysis, ferroptosis was also associated with immunotherapy of tumors. The effector function of CD8^+^ T cells in the tumor microenvironment can be restored or increased by cancer immunotherapy [51]. Wang et al. found that immunotherapy-activated CD8^+^ T cells increase ferroptosis-specific lipid peroxidation in cancer cells, and enhanced ferroptosis facilitates the antitumor efficacy of immunotherapy [26, 52]. In addition, some iron-rich cancers (such as breast cancer and HCC) or tumors rich in reactive oxygen species (lung cancer) are particularly sensitive to drugs that promote ferroptosis. So, some ferroptosis-related antitumor agents have been identified. Such as the ferroptosis inducer sulfasalazine which targets SLC7A11 might be effective against tumor cells overexpressing SLC7A11 [49, 53]. In addition, Sulfasalazine was discovered to promote ferroptosis by suppressing cysteine uptake and GPX4 synthesis [54]. Cancer-associated fibroblasts (CAFs)-secreted miR-522 inhibits ferroptosis and facilitates acquired chemotherapy resistance in gastric cancer [27]. Some novel approaches have also attempted to treat cancer by inducing ferroptosis, such as Fe3 O4 -SAS @ PLT, biomimetic magnetic nanoparticles, constructed from mesoporous magnetic nanoparticles (Fe3O4) loaded with salazosulfapyridine (SAS) and platelet (PLT) membranes camouflaged and triggered via inhibition of the glutamate-cystine reverse transport system Xc pathway in ferroptosis. Jiang et al. reported that Fe3O4-SAS@PLT-mediated ferroptosis enhances the therapeutic efficacy of PD-1 immune checkpoint blockade and achieves sustained tumor elimination in a mouse model of metastatic tumors [55]. Furthermore, ultra-small nanoparticles were found to induce ferroptosis and suppress tumor growth in nutrient-deficient cancer cells [56]. Several clinical trials are currently underway to test the efficacy and safety of ferroptosis inducers or anticancer drugs with ferroptosis-inducing activity in cancer patients, alone or in combination with conventional therapy, such as NCT02559778, NCT04092647, NCT04205357, NCT03247088, et al. [57].

### Necroptosis in cancer

Necroptosis, another form of ICD, was reported by Degerev et al. [58] in 2005 and consists mainly of specific death receptors (DR) such as FAS and TNFR1 or TLR3 recognizing unfavorable signals from the intracellular and extracellular microenvironment to trigger necroptosis [59, 60]. Since then, there has been a proliferation of research on “necroptosis in cancer”. Han et al. first reported in 2007 that the small molecule compound- shikonin (a naturally occurring naphthoquinone), circumvents cancer drug resistance by inducing necroptosis [61]. In 2016, Aaes et al. first identified necroptosis in tumors as a form of immunogenic cell death and necroptosis tumor cells can induce dendritic cell maturation, cytotoxic T cell crossover initiation, and IFN-γ production [62]. Also, several studies have shown that the necroptosis-induced inflammatory response may contribute to anticancer therapy. Necroptotic cancer cells release interleukin-1a (IL-1a), which activated dendritic cells (DCs) to produce IL-12 (a cytokine essential for antitumor response) [63]. Polyinosinic acid (polyI:C)-induced necroptosis can support in vivo immune effector-mediated tumor elimination [64]. Another study showed that the necrosome can promote pancreatic cancer through C-X-C motif chemokine ligand 1 (CXCL1) and Mincle-induced immunosuppression [65]. In addition, necrosis is also involved in the metastatic process of cancer. Strilic et al. found that human and mouse tumor cells induce necroptosis of endothelial cells, thereby promoting tumor cell extravasation and metastasis. Further studies revealed that tumor cell-induced endothelial cell necrosis resulting in extravasation and metastasis needed amyloid precursor proteins expressed through tumor cells and their receptor, death receptor 6 (DR6), on endothelial cells as the main mediators of these actions [66]. MLKL, as an executor of necroptosis, may influence cancer progression and metastasis via necroptosis-dependent and non-dependent functions [30]. Shikonin significantly reduces osteosarcoma metastasis by inducing receptor-interacting protein 1 (RIPK1)- and RIPK3-dependent necroptosis [67]. Similar effects of shikonin have been reported in gliomas, and Huang et al. found shikonin kills C6 and U87 glioma cells through necroptosis mediated by RIPK-1 [68]. Shikonin was also found to reduce the growth of doxorubicin-resistant prostate cancer cells mainly through necroptosis [69]. The cross-priming process of stimulating naive cytotoxic CD8+ T cells is required for the immune process against most tumors [70]. Necroptotic cells activate adaptive immunity by supplying antigenic and inflammatory stimuli to DCs, which in turn activate CD8+ T cells and anti-tumor immunity. RIPK1 and NF-κB signaling in dying cells dictate cross-priming of CD8+ T cells [71]. Another study confirmed that RIPK1 and RIPK3 may be highly expressed in tumor-reactive T cells and undergo necrosis upon restimulation of the T cell receptor (TCR) with cognate antigen, which can be inhibited via RIPK1 inhibition [72, 73]. Snyder et al. found that infusion of cells undergoing necroptosis into mouse tumors directs killer T cells to attack malignant tumors and slow their growth [74]. Baik et al. found that Z-DNA-binding protein 1 (ZBP1) mediates tumor necroptosis in breast cancer [29]. Recent studies have shown that the RNA editing enzyme ADAR1 is a novel determinant of ICB therapeutic resistance that blocks the ICB response by inhibiting immunogenic double-stranded RNA (dsRNA), and that depletion or mutation of ADAR1 leads to Z-RNA accumulation and activation of the Z-RNA sensor ZBP1, ultimately leading to RIPK3-mediated necroptosis. This finding provides an easily translatable pathway for the immune response in ICB-resistant human cancers. The investigators screened the small molecule compound CBL0137 to activate ZBP1-dependent nuclear necroptosis. CBL0137 in combination with PD-1 antibody significantly induced melanoma regression in mice, indicating the great potential of CBL0137 in immunotherapy [75]. In addition, CBL0137 has been shown to be well tolerated in phase 1 clinical trials in other studies, implying that CBL0137 has good prospects for clinical application [76].

### Pyroptosis in cancer

Pyroptosis is primarily mediated via inflammasomes to induce various caspases, such as CASP1, CASP4, CASP5, and CASP11 (mouse) [77, 78]. The most important pathway through which pyroptosis occurs is that inflammatory caspases can direct cut and activate the gasdermin D (GSDMD), mediating plasma membrane pores formation and leading to cell death [79]. In 2015, Shi et al. identified gasdermin D (GSDMD) and proposed that GSDMD is a central mediator downstream of caspase-1/4/5/11 that causes focal hypoplasia and is crucial for IL-1β secretion [10]. Pyroptosis plays a twofold role in cancer development, promoting the tumor or leading to regress, depending on the environment in which the tumor cells are located. As mentioned above, pyroptosis activation can lead to the release of IL-1 and IL-1β, which can promote cancer development in several ways. Our previous study discovered that the gasdermin family members are involved in the occurrence and development of HCC, the expression of GSDMB and GSDMD is significantly higher in HCC tumor samples compared with corresponding normal samples [80]. The expression of Caspase-1, IL-1β, and IL-18 in HCC tissues was lower than that in corresponding adjacent normal tissues [81]. Study indicates that the absent in melanoma 2 (AIM2) inflammasome mediate pyroptosis acts a crucial role in radiation gastrointestinal syndrome [82]. AIM2 was decreased in ~67.4% of colorectal cancer (CRC) cells and was directly absent in 9.18% of CRC cells. Dihlmann et al. reported that after adjusting for clinicopathological characteristics, complete lack of AIM2 expression was associated with significantly increased overall and disease-specific mortality compared to AIM2-positive tumor samples, suggesting that lack of AIM2 expression is strongly related to poor outcomes in CRC. Thus, these data strongly confirm the protective role of AIM2 against colorectal tumor progression [83]. Tan et al. found that high-mobility group box protein 1 (HMGB1), as a pro-inflammatory factor released from GSDME-mediated inflammatory epithelial cells, induces CRC proliferation via the ERK1/2 pathway [84]. Hergueta-Redondo et al. found that GSDMB-2 induces invasion, tumor progression, and metastasis in MCF7 breast cancer cells, and overexpression of GSDMB predicts low responsiveness to erb-b2 receptor tyrosine kinase 2 (HER2)-targeted therapy in HER2-positive breast cancer [85]. In addition, DFNA5 methylation has been identified to be related to lymph node metastasis [86]. Also, decreased DNFA5 was related to increased etoposide resistance in melanoma [87]. In the field of lung cancer, Xi et al. discovered that GSDMD facilitated cytotoxic T lymphocyte-mediated lung squamous and lung adenocarcinoma killing [88]. In addition, the mechanisms of certain antitumor drugs include pyroptosis. Wang et al. reported that chemotherapeutic drugs induce high levels of GSDME pyroptosis in tumor cells due to caspase-3 activation, which exerts its suppression oncogenic effect [89]. GSDME is also involved in mediating focal hypoplasia and caspase-3/9 activation downstream of the ROS/JNK/BAX mitochondrial apoptosis pathway induced by lopressor in CAC cells [90]. Wu et al. found PLK1 kinase inhibitor increases chemosensitivity to cisplatin via triggering pyroptosis in esophageal squamous cell carcinoma [91]. Paclitaxel and cisplatin induce apoptosis via caspase-3/GSDME activation in A549 lung cancer cells [92]. Erkes et al. had reported that BRAF mutation and MEK inhibitors regulation of the tumor immune microenvironment through cellular pyroptosis [93]. The results of an interesting study indicate that PD-L1 mediates the atypical pyroptosis in cancer cells through GSDMC/caspase-8, leading to tumor necrosis [32]. Zhang et al. reported GSDME suppresses tumor growth via activating anti-tumor immunity [31]. Furthermore, granzyme A will cleave GSDMB to induce pyroptosis in target cells [94]. The relationship between pyroptosis and antitumor immunity is uncertain and deserves further study. Besides, it has been shown that certain anti-cancer drugs cause tumor cell death by pyroptosis. Such as, Shao et al. first reported that chemotherapeutic agents, including topotecan and doxorubicin, could treat lung cancer by caspase-3 cleavage of GSDME [89]. Furthermore, an increasing number of chemically targeted drugs, agents and natural products are now being found to cause pyroptosis in various types of cancer, many of which have antitumor effects. Such as α-NETA, Cisplatin, Sorafenib, Lobaplatin, et al. [90, 95–97]. Furthermore, Chimeric Antigen Receptor T-Cell induce primary B leukemia cells to activate caspase-3 cleavage of GSDME and ultimately lead to pyroptosis [98]. Recently, researchers designed a novel chimeric costimulatory transforming receptor (CCCR) that can rapidly induce GSDME-dependent lung cancer pyroptosis and effectively enhances antitumor activity by reversing PD1 immunosuppression [99].

### Cuproptosis in cancer

Cuproptosis is a regulated cell death triggered via excess Cu^2+^ that was recently reported by Tsvetkov et al. [11]. Because copper ions might be involved in the activation of signaling pathways related to cell proliferation, the role of copper in cancer progression has been a research direction. Studies have shown that cancer cells have a higher demand for copper than healthy resting cells [100]. Such as breast cancer [101], thyroid cancer [102], oral cancer [103], ovarian cancer [104], pancreatic cancer [105], gallbladder [106], et al. Besides, studies have indicated that copper can be involved in cancer development through promoting angiogenesis, cell proliferation, and metastasis. The addition of 20 μM CuSO_4_ to drinking water accelerates tumor growth in a pancreatic islet cell carcinoma mouse model [107]. In addition, daily administration of CuSO_4_ can enhance tumor growth in a rat model of chemically induced mammary carcinogenesis [108]. Interestingly, copper can also drive carcinogenesis to the autophagic kinase ULK1/2 [109]. More importantly, the concept that copper is angiogenic was first introduced by McAuslan. He discovered that copper salts can induce endothelial cell migration, which is an early step in angiogenesis [110]. This hypothesis was subsequently tested in in vivo and in vitro experiments [111, 112]. Copper deficiency has been demonstrated to inhibit the transcriptional activity of NF-κB, thereby suppressing pro-angiogenic factors expressions, such as IL-1α, IL-6, IL-8, and vascular endothelial growth factor (VEGF) [113]. Disulfiram (DSF) has been indicated to be able to induce cuproptosis [11]. In vitro researches have demonstrated that DSF has antitumor activity in a variety of cancers when combined with copper ions [Cu(II) [114]. Preclinical researches have demonstrated that the combination of DSF and Cu (II) specifically targets and kills aldehyde dehydrogenase (ALDH)^+^ cancer stem cells, decreasing the risk of tumor recurrence [115, 116]. Since cuproptosis is only recently discovered, its research in the field of cancer is only at an initial exploratory stage, and more in-depth studies are needed subsequently, and since there are no reliable biomarkers, this will be a long-term bottleneck limiting the development of clinical applications targeting cuproptosis.

## Limitation

This is the first bibliometric analysis of “ferroptosis in cancer”, “necroptosis in cancer”, “pyroptosis in cancer”, and “cuproptosis in cancer” using VOSviewer software, but there are still some limitations. First of all, The literature we obtained was from January 1, 2012, to October 31, 2022. However, the literature in web of science core collection (WOSCC) is updated all the time and the search results of this research are somewhat diverse from the actual number of included literature. Second, during the literature search, some keywords are not fully included in the analysis and the results might have been influenced by incomplete keyword extraction. Finally, this research includes articles and reviews, and the quality of the selected literature varies, perhaps reducing the credibility of the overall analysis. However, bibliometric analysis based on literature undoubtedly provides scholars with a quick overview of research topics, research hot spots, and trends in the field of “ferroptosis in cancer”, “necroptosis in cancer”, “pyroptosis in cancer”, and “cuproptosis in cancer”. WOSCC is the main database for bibliometric analysis and we believe that this work is representative of the general situation and general trends in “ferroptosis in cancer”, “necroptosis in cancer”, “pyroptosis in cancer”, and “cuproptosis in cancer”.

## Conclusion

This study presents a bibliometric analysis of the current state of research on “ferroptosis in cancer”, “necroptosis in cancer”, “pyroptosis in cancer”, and “cuproptosis in cancer”, four of the hottest research areas in cancer research, with an increasing number of scholars, organizations, and countries involved and a large number of high-quality publications. Visual analysis using VOSviewer software shows that research on “ferroptosis in cancer”, “necroptosis in cancer”, and “pyroptosis in cancer” are increasing year by year. Especially after 2020, the research in these three areas has skyrocketed and the research on “cuproptosis in cancer” started in 2022 and is still in the initiation phase. Worldwide, China and the United States are leaders in all four of these research areas. Cooperation and communication between different scholars as well as countries and organizations need to be strengthened in the future. Together, they can promote the development of related research fields and explore more research hotspots. Currently, research on PCD in cancer is focused on the mechanisms, the crosstalk of different types of PCD, and their role in cancer, which will be the hot spots of future research. Further exploration of inhibitors of different PCDs and their targeted therapies are potential treatment options for cancer, but more direct clinical evidence as well as higher-level clinical trials remain to be explored. Further clarification of the mechanisms of crosstalk between these PCDs may provide effective cancer treatments. And the role of different types of PCDs, especially the novel ones discovered, in cancer can be expected to remain a hot topic of research in the cancer field for quite some time to come.

## Materials and methods

### Data collection

The keywords “ferroptosis and cancer”; “necroptosis and cancer”, “pyroptosis and cancer”, “cuproptosis and cancer” were indexed in WOSCC, respectively. Articles from January 1, 2012 to October 31, 2022 were retrieved, and search themes were as follows: “TS = (ferroptosis)”, AND “TS = (cancer) OR TS = (tumor)”. “TS = (necroptosis)”, AND “TS = (cancer) OR TS = (tumor)”. TS = (pyroptosis)”, AND “TS = (cancer) OR TS = (tumor)”. TS = (cuproptosis)”, AND “TS = (cancer) OR TS = (tumor)”. A total of 3302 (ferroptosis in cancer), 2233 (necroptosis in cancer), 1445 (pyroptosis in cancer), and 109 (cuproptosis in cancer) references were exported and retrieved records will be exported as all references and records, saved as plain text files and stored in savedrecs_text format.

### Data analysis

First, the general information of the literature, including year of publication, country, organization, journal and author, was initially analyzed through the analysis and search results in WOSCC. Then, the VOSviewer software (Version 1.6.18) was utilized to conduct bibliometric and visual analysis,

Type of analysis including Co-authorship, Co-occurrence, Citation, Bibliographic coupling, and Co-citation. Co-authorship analysis:The relevance of items was identified by their number of co-authored documents. Co-occurrence analysis: The relevance of projects is identified according to the number of documents in which they occur together. Citation analysis: The relevance of items is identified according to the number of times they cite each other. Bibliographic coupling analysis: The relevance of items is determined based on the number of references they share. Co-citation analysis: The relevance of itens is identified according to the number of times they are cited together. The counting method was used for ranking order, and association strength was applied normalization in the VOSviewer software. We utilized microsoft office excel 2019 to analyze the trend of the number of publications per year and visualized the results by GraphPad Prism 8 software.

## Supplementary information

Table S1

Supplementary Figure legends

Figure S1

Figure S2

Figure S3

Figure S4

Figure S5

Figure S6

Figure S7

Figure S8

Figure S9

Figure S10

## Data Availability

The data that support the findings of this study are available from Web of Science Core Collection.
