# Supplementary material for: A bibliometric analysis of ferroptosis, necroptosis, pyroptosis, and cuproptosis in cancer from 2012 to 2022
Source: Cell Death Discov. 2023 Apr 15;9:129. doi: 10.1038/s41420-023-01421-1 (PMC10105750; doi:10.1038/s41420-023-01421-1)
Supplement: Supplementary file 1 — Table S1 [file 41420_2023_1421_MOESM1_ESM.docx]

Table S1. Top 10 keywords related to ferroptosis, necroptosis, pyroptosis, and cuproptosis in cancer

| **Type** | **Rank** | **Keyword** | **Occurrences** | **Avg. pub. year** | **Avg. citations** |
| --- | --- | --- | --- | --- | --- |
| Ferroptosis in cancer | 1 | Ferroptosis | 4402 | 2021 | 20 |
|  | 2 | Prognosis | 538 | 2022 | 5 |
|  | 3 | Apoptosis | 525 | 2020 | 27 |
|  | 4 | Cancer | 504 | 2020 | 33 |
|  | 5 | Iron | 506 | 2020 | 48 |
|  | 6 | Lipid peroxidation | 458 | 2021 | 23 |
|  | 7 | Autophagy | 483 | 2020 | 43 |
|  | 8 | Cell death | 404 | 2020 | 38 |
|  | 9 | Gpx4 | 325 | 2021 | 23 |
|  | 10 | Immunotherapy | 336 | 2022 | 10 |
|  |  |  |  |  |  |
| Necroptosis  in cancer | 1 | Necroptosis | 878 | 2019 | 29 |
|  | 2 | Apoptosis | 462 | 2018 | 33 |
|  | 3 | Autophagy | 183 | 2018 | 50 |
|  | 4 | Cell death | 146 | 2019 | 26 |
|  | 5 | Cancer | 106 | 2019 | 37 |
|  | 6 | Ferroptosis | 104 | 2020 | 31 |
|  | 7 | Inflammation | 102 | 2019 | 30 |
|  | 8 | Pyroptosis | 82 | 2020 | 29 |
|  | 9 | Necrosis | 81 | 2017 | 63 |
|  | 10 | Mlkl | 74 | 2019 | 50 |
|  |  |  |  |  |  |
| Pyroptosis  in cancer | 1 | Pyroptosis | 704 | 2021 | 15 |
|  | 2 | Apoptosis | 152 | 2020 | 26 |
|  | 3 | Prognosis | 125 | 2022 | 5 |
|  | 4 | Inflammasome | 100 | 2020 | 32 |
|  | 5 | Immunotherapy | 87 | 2022 | 6 |
|  | 6 | Necroptosis | 87 | 2020 | 39 |
|  | 7 | Inflammation | 83 | 2020 | 15 |
|  | 8 | Ferroptosis | 79 | 2021 | 18 |
|  | 9 | Autophagy | 77 | 2020 | 26 |
|  | 10 | Tumor microenvironment | 71 | 2022 | 8 |
|  |  |  |  |  |  |
| Cuproptosis  in cancer | 1 | Cuproptosis | 79 | 2022 | 1 |
|  | 2 | Prognosis | 33 | 2022 | 0 |
|  | 3 | Immunotherapy | 21 | 2022 | 0 |
|  | 4 | Tumor microenvironment | 14 | 2022 | 0 |
|  | 5 | LncRNA | 13 | 2022 | 0 |
|  | 6 | Prognostic signature | 9 | 2022 | 0 |
|  | 7 | Lung adenocarcinoma | 7 | 2022 | 0 |
|  | 8 | Overall survival | 7 | 2022 | 3 |
|  | 9 | Prognostic model | 7 | 2022 | 1 |
|  | 10 | Drug sensitivity | 6 | 2022 | 0 |
