# Supplementary material for: A bibliometric analysis of ferroptosis, necroptosis, pyroptosis, and cuproptosis in cancer from 2012 to 2022
Source: Cell Death Discov. 2023 Apr 15;9:129. doi: 10.1038/s41420-023-01421-1 (PMC10105750; doi:10.1038/s41420-023-01421-1)
Supplement: Supplementary file 2 — Supplementary Figure legends [file 41420_2023_1421_MOESM2_ESM.docx]

**Figure S1. Distribution of publications from different countries/regions in the Co-author ship analysis.** (A) ferroptosis in cancer; (B) necroptosis in cancer; (C) pyroptosis in cancer; (D) cuproptosis in cancer. Each circle in the figure represents a country/region, and the size of the circle indicates the number of publications output in that country. The lines between the circles indicate cooperation between countries; the wider the line, the closer the cooperation. Different colors represent different clusters.

**Figure S2. Distribution of publications from different countries/regions in** **Citation analysis.** (A) ferroptosis in cancer; (B) necroptosis in cancer; (C) pyroptosis in cancer; (D) cuproptosis in cancer.

**Figure S3. Distribution of publications from different countries/regions in** **bibliographic coupling analysis.** (A) ferroptosis in cancer; (B) necroptosis in cancer; (C) pyroptosis in cancer; (D) cuproptosis in cancer.

**Figure S4. Distribution of publications from different organizations in the Co-author ship analysis.** (A) ferroptosis in cancer; (B) necroptosis in cancer; (C) pyroptosis in cancer; (D) cuproptosis in cancer.

**Figure S5. Distribution of publications from different organizations in** **Citation analysis.** (A) ferroptosis in cancer; (B) necroptosis in cancer; (C) pyroptosis in cancer; (D) cuproptosis in cancer.

**Figure S6. Distribution of publications from different organizations in** **bibliographic coupling analysis.** (A) ferroptosis in cancer; (B) necroptosis in cancer; (C) pyroptosis in cancer; (D) cuproptosis in cancer.

**Figure S7. VOSviewer visualization map of Co-authorship authors.** (A) ferroptosis in cancer; (B) necroptosis in cancer; (C) pyroptosis in cancer; (D) cuproptosis in cancer. Each circle indicates an author, the size of the circle indicates the number of articles published by that author, the larger the circle, the more articles published in the field, the lines between the circles indicate the connections between authors, and the connection networks of different colors indicate the clusters of collaboration between different authors. Different colors represent different clusters.

**Figure S8. VOSviewer visualization map of Citation Authors.** (A) ferroptosis in cancer; (B) necroptosis in cancer; (C) pyroptosis in cancer; (D) cuproptosis in cancer. Each circle indicates an author, the size of the circle indicates the number of citations of that author's published articles, the larger the circle, the higher the number of citations, the lines between the circles indicate the connection between authors, and the connection networks of different colors indicate the clusters of cooperation between different authors. Different colors represent different clusters.

**Figure S9. VOSviewer visualization map of Citation Source.** (A) ferroptosis in cancer; (B) necroptosis in cancer; (C) pyroptosis in cancer; (D) cuproptosis in cancer. Each circle indicates a journal, the size of the circle indicates the number of published articles in that journal, the larger the circle, the higher the number of publications, the lines between the circles indicate the connection between journals, and the connection networks of different colors indicate the clusters of cooperation between different journals. Different colors represent different clusters.

**Figure S10. VOSviewer visualization map of keywords co-occurrence analysis.** (A) ferroptosis in cancer; (B) necroptosis in cancer; (C) pyroptosis in cancer; (D) cuproptosis in cancer. Each circle indicates a keyword, the size of the circle indicates the number of occurrences, the larger the circle, the higher the number of occurrences, the lines between the circles indicate the connection between keyword, and the connection networks of different colors indicate the clusters of cooperation between different keywords. Different colors represent different clusters.
