# Supplementary figures and images for: A bibliometric analysis of ferroptosis, necroptosis, pyroptosis, and cuproptosis in cancer from 2012 to 2022
Source: Cell Death Discov. 2023 Apr 15;9:129. doi: 10.1038/s41420-023-01421-1 (PMC10105750; doi:10.1038/s41420-023-01421-1)

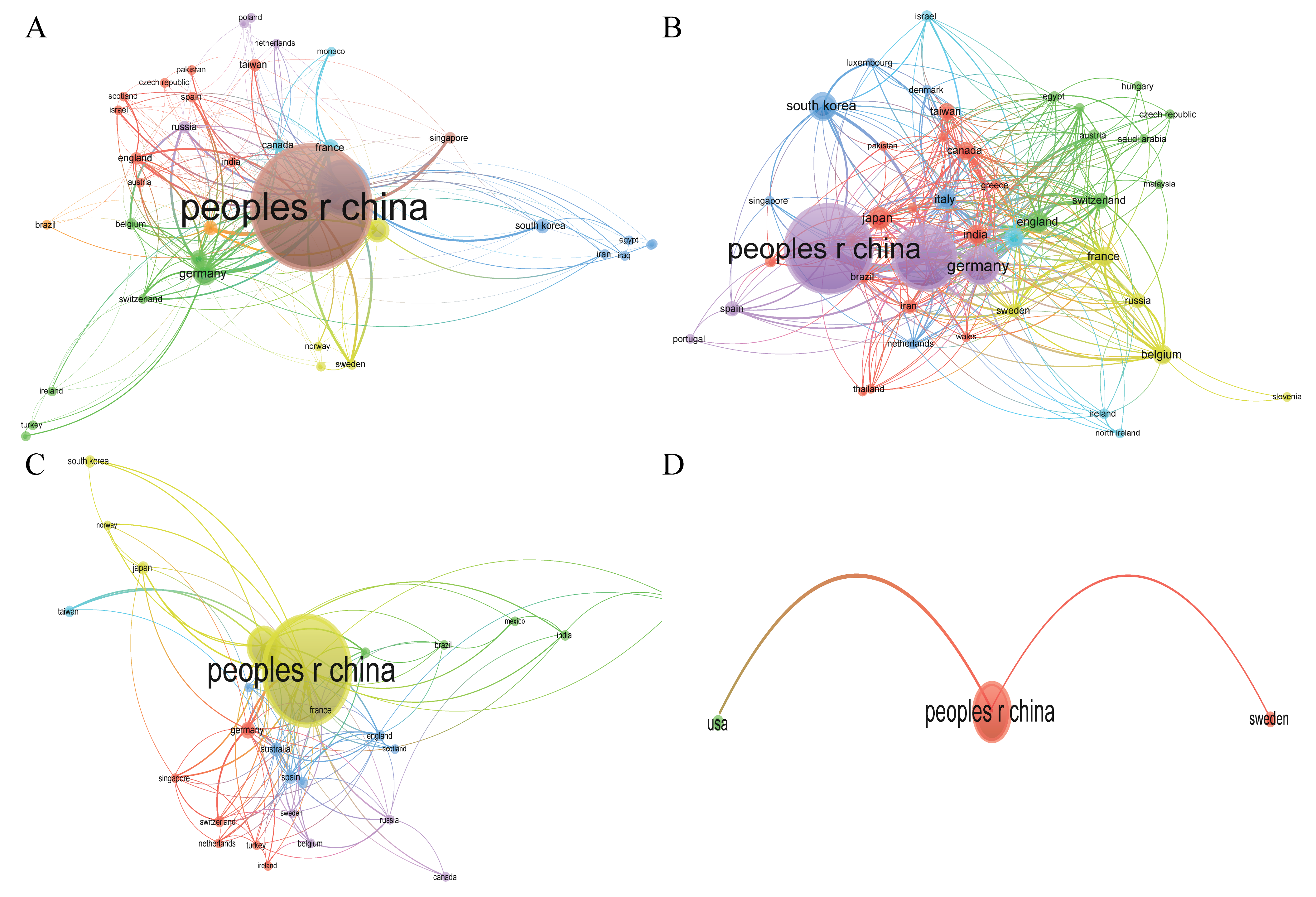

Supplement: Supplementary file 3 — Figure S1 [file 41420_2023_1421_MOESM3_ESM.tif]

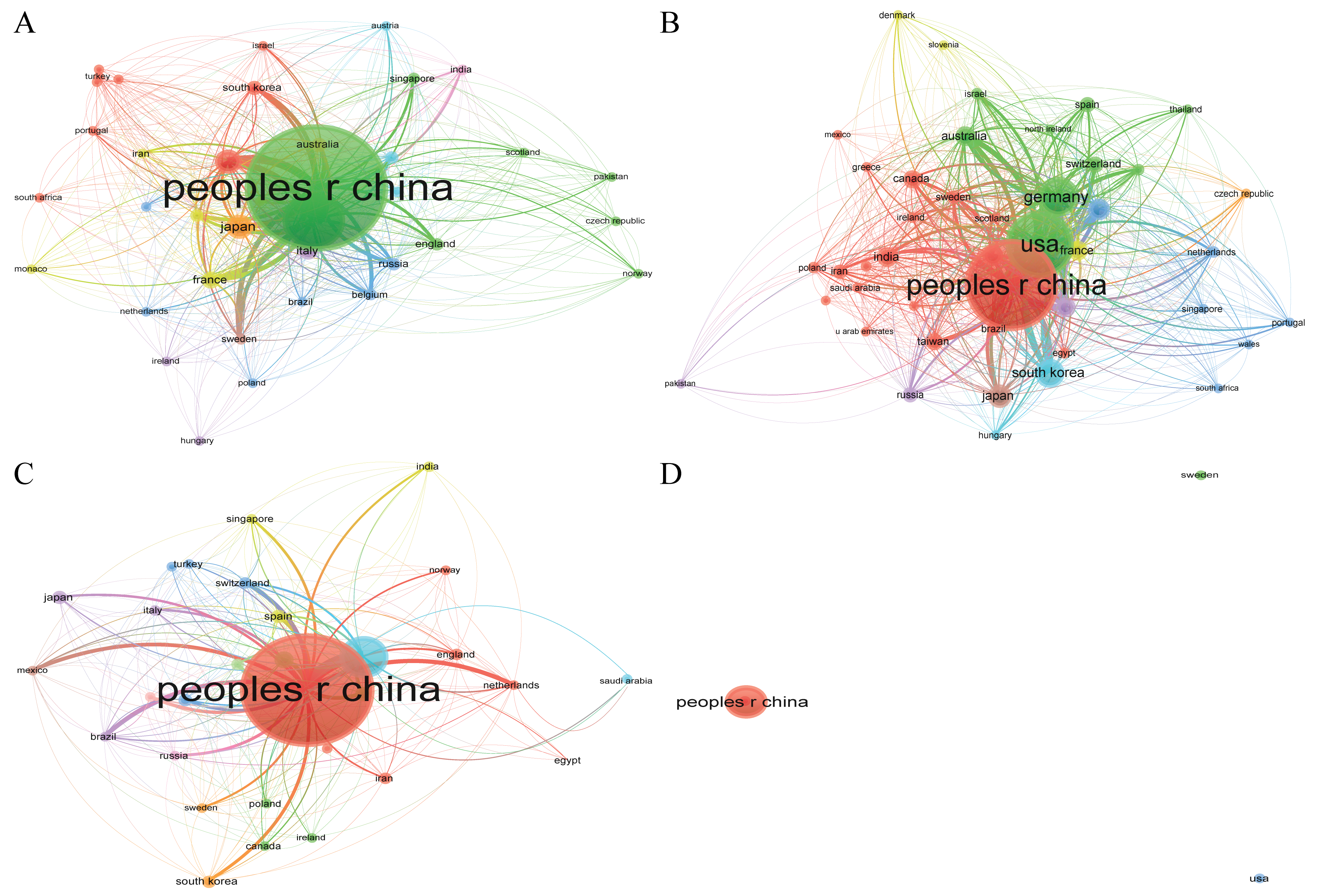

Supplement: Supplementary file 4 — Figure S2 [file 41420_2023_1421_MOESM4_ESM.tif]

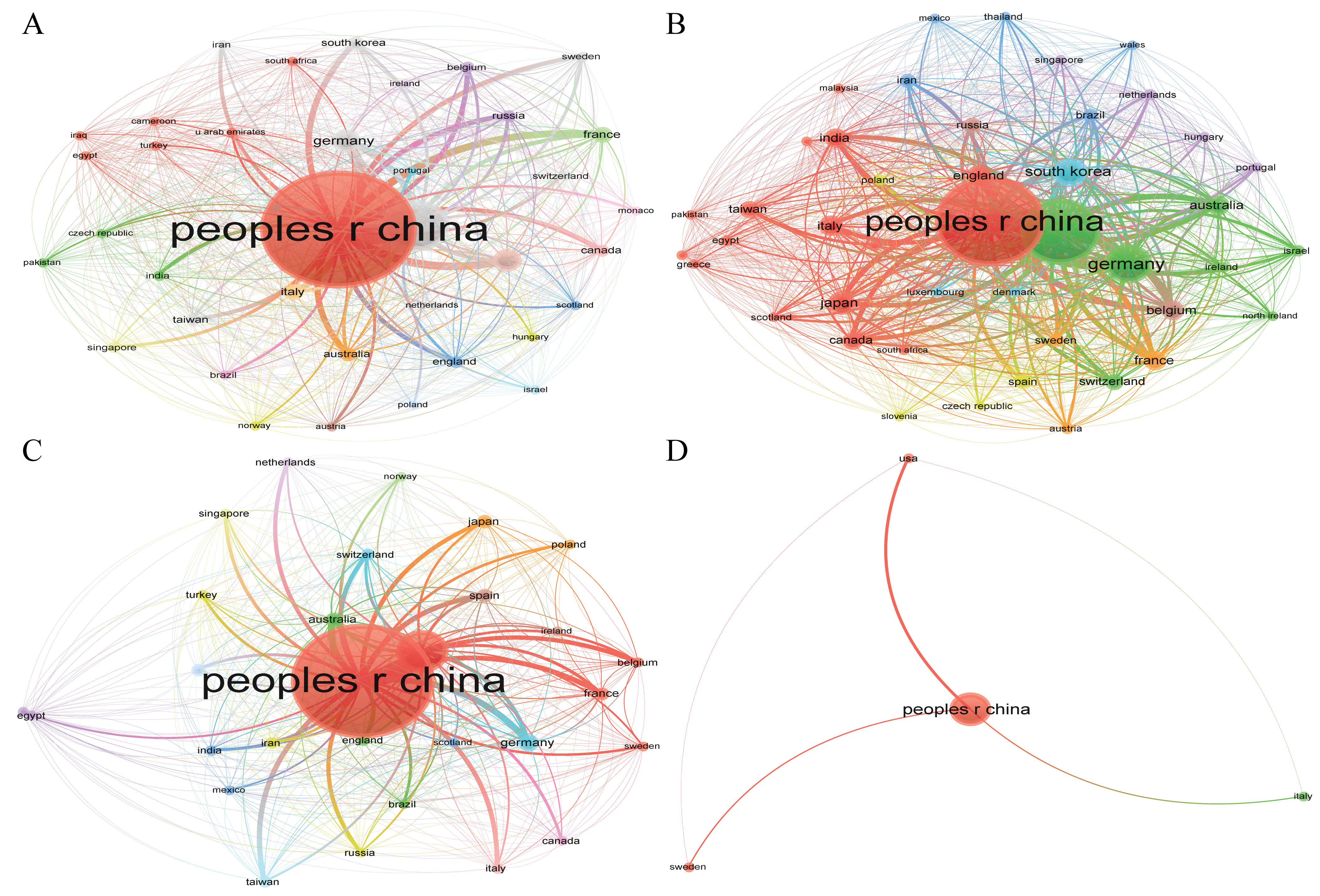

Supplement: Supplementary file 5 — Figure S3 [file 41420_2023_1421_MOESM5_ESM.tif]

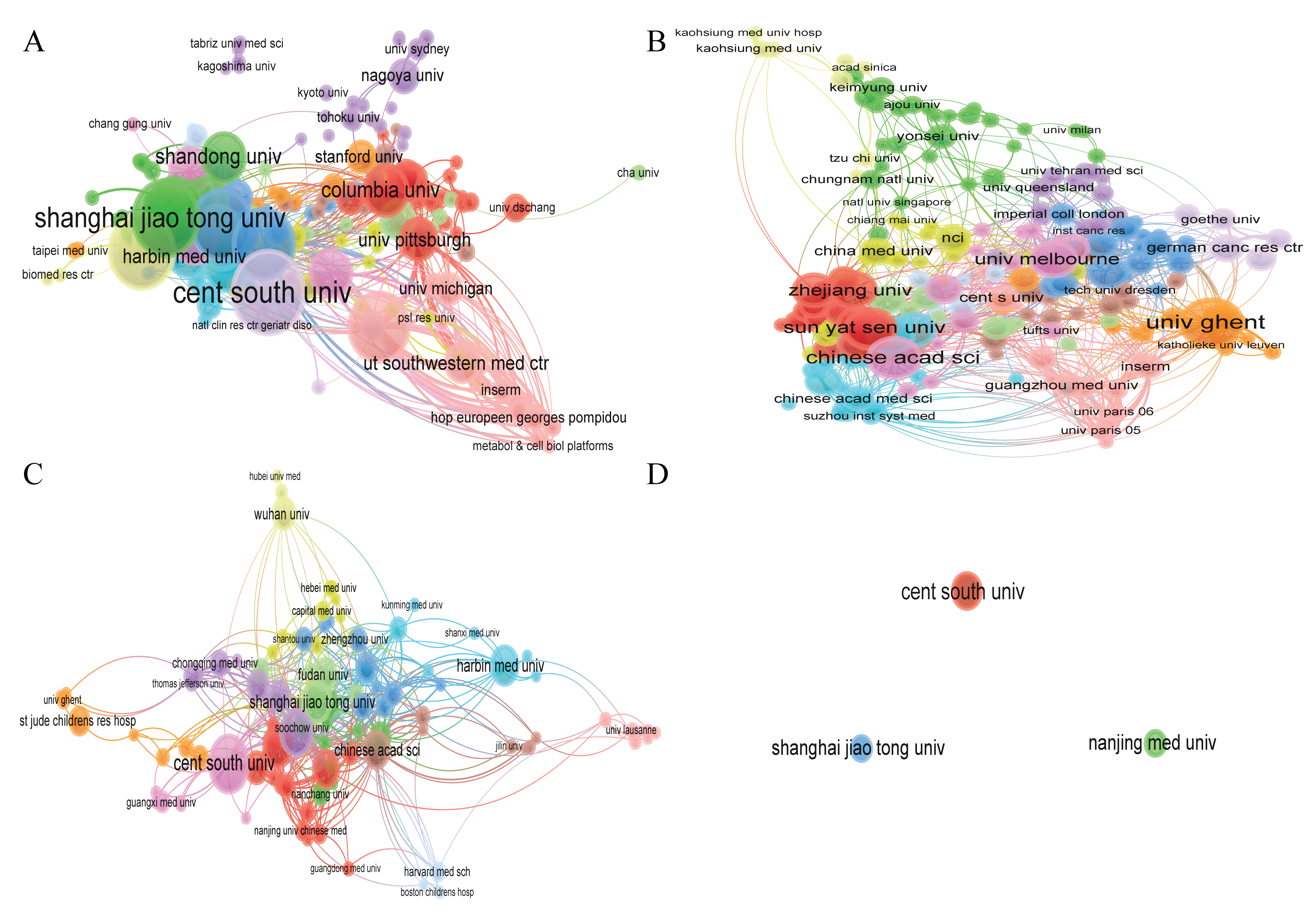

Supplement: Supplementary file 6 — Figure S4 [file 41420_2023_1421_MOESM6_ESM.tif]

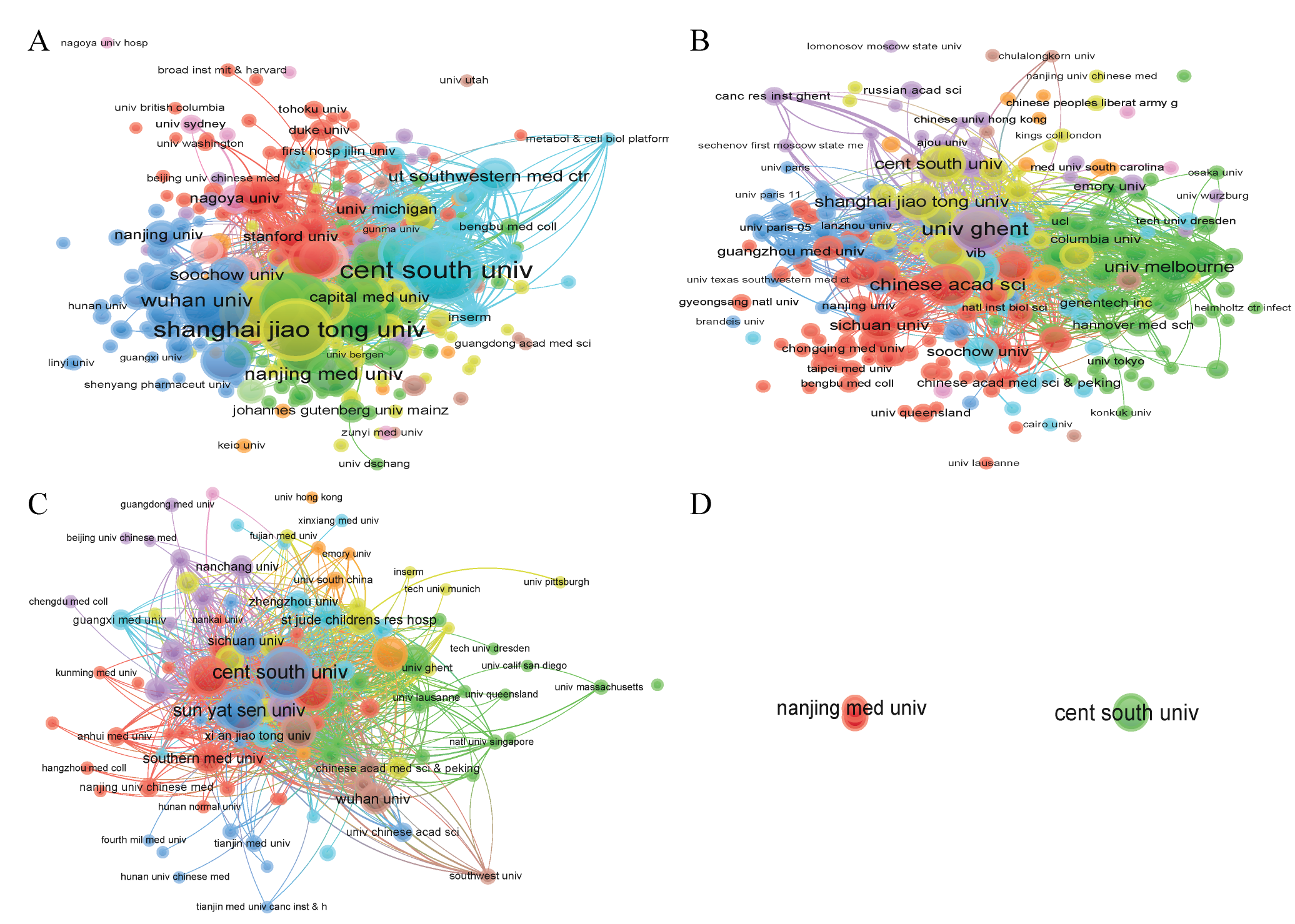

Supplement: Supplementary file 7 — Figure S5 [file 41420_2023_1421_MOESM7_ESM.tif]

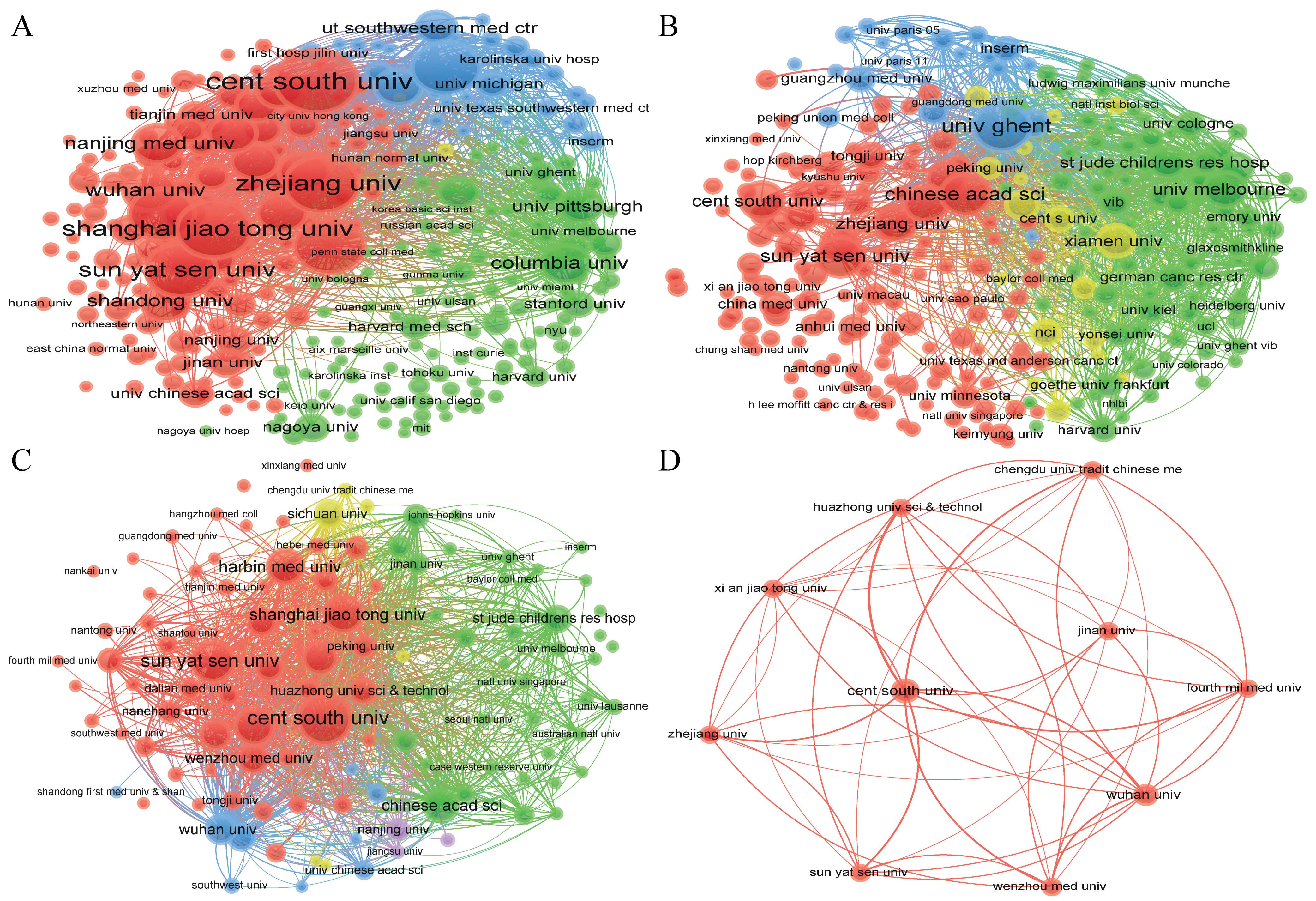

Supplement: Supplementary file 8 — Figure S6 [file 41420_2023_1421_MOESM8_ESM.tif]

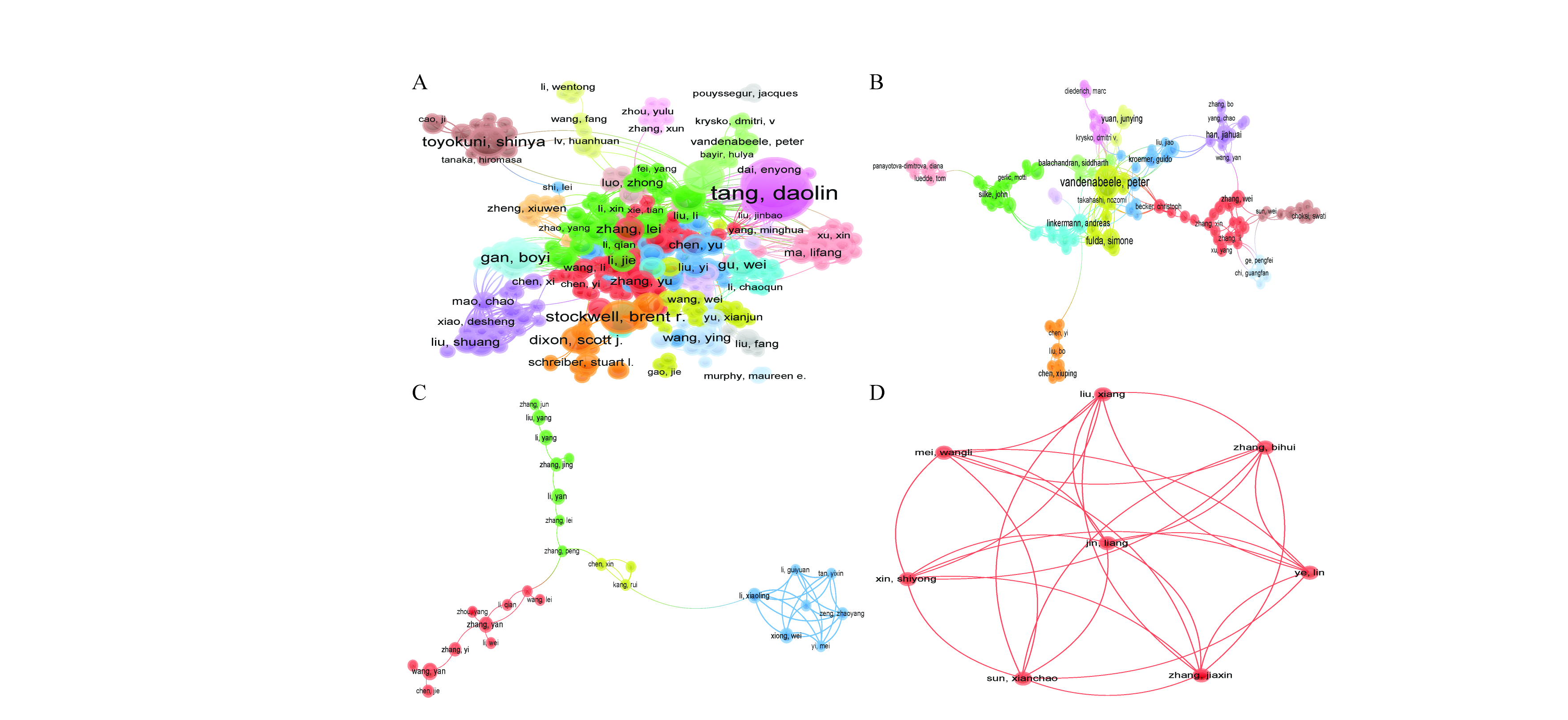

Supplement: Supplementary file 9 — Figure S7 [file 41420_2023_1421_MOESM9_ESM.tif]

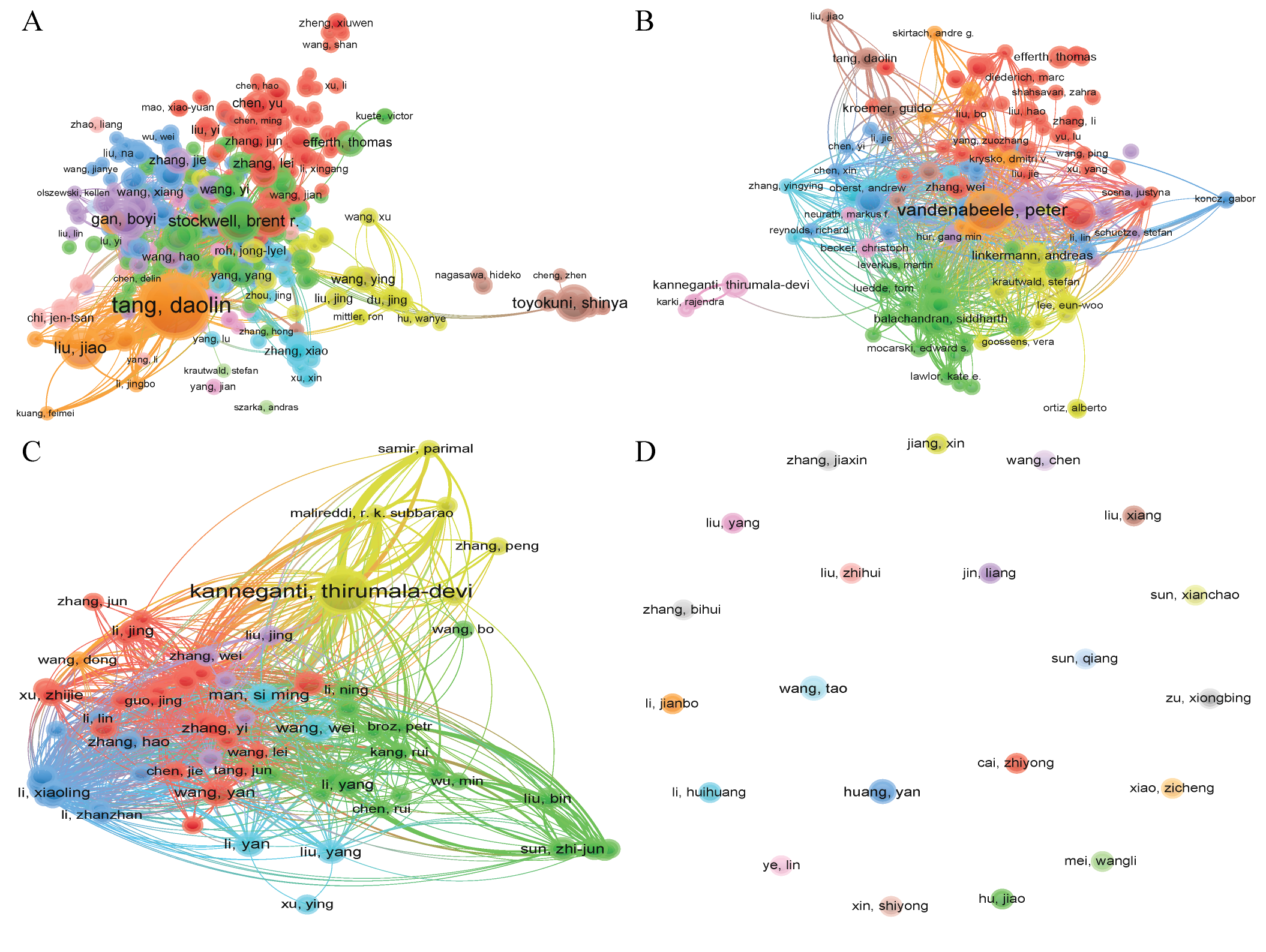

Supplement: Supplementary file 10 — Figure S8 [file 41420_2023_1421_MOESM10_ESM.tif]

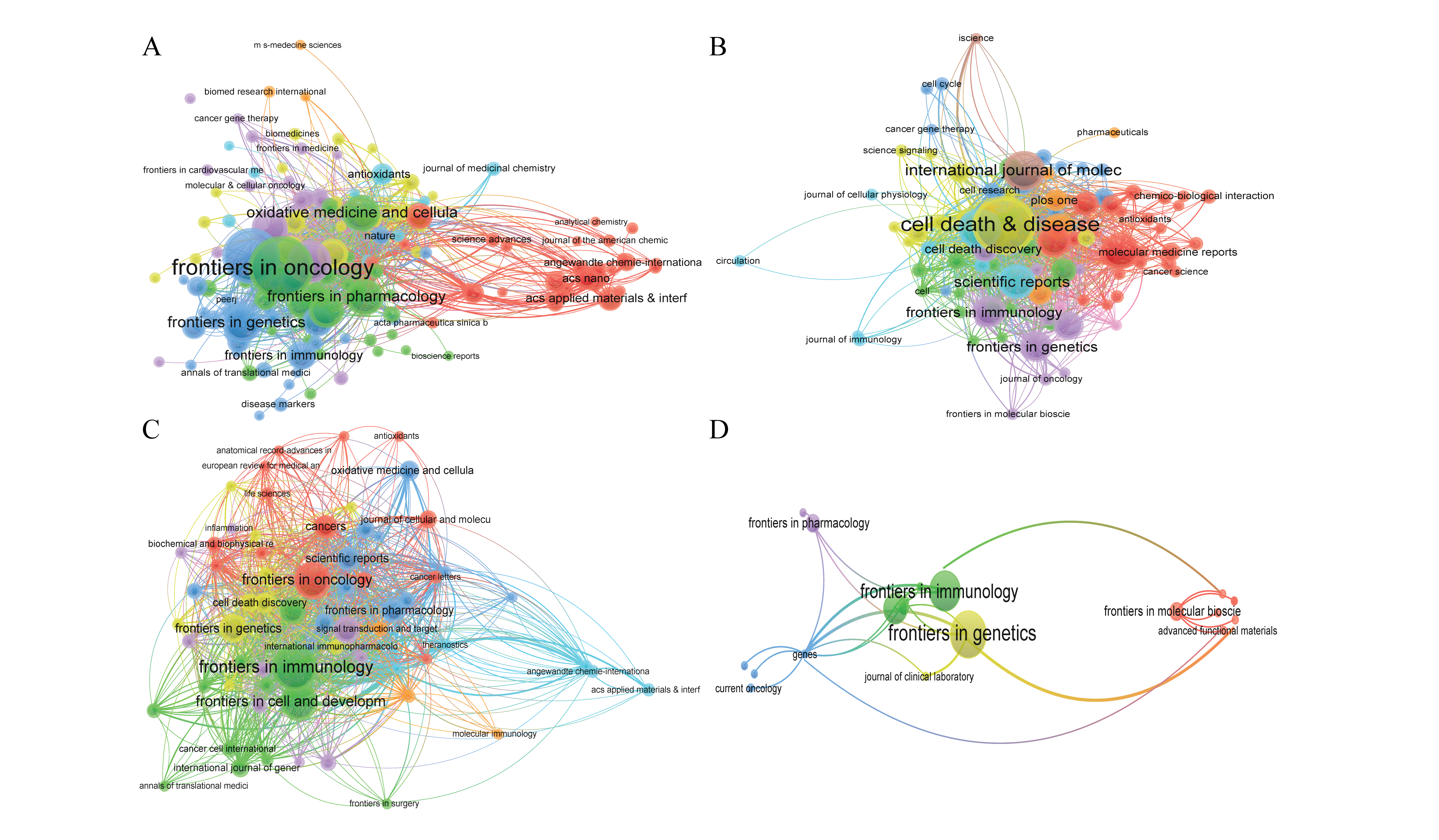

Supplement: Supplementary file 11 — Figure S9 [file 41420_2023_1421_MOESM11_ESM.tif]

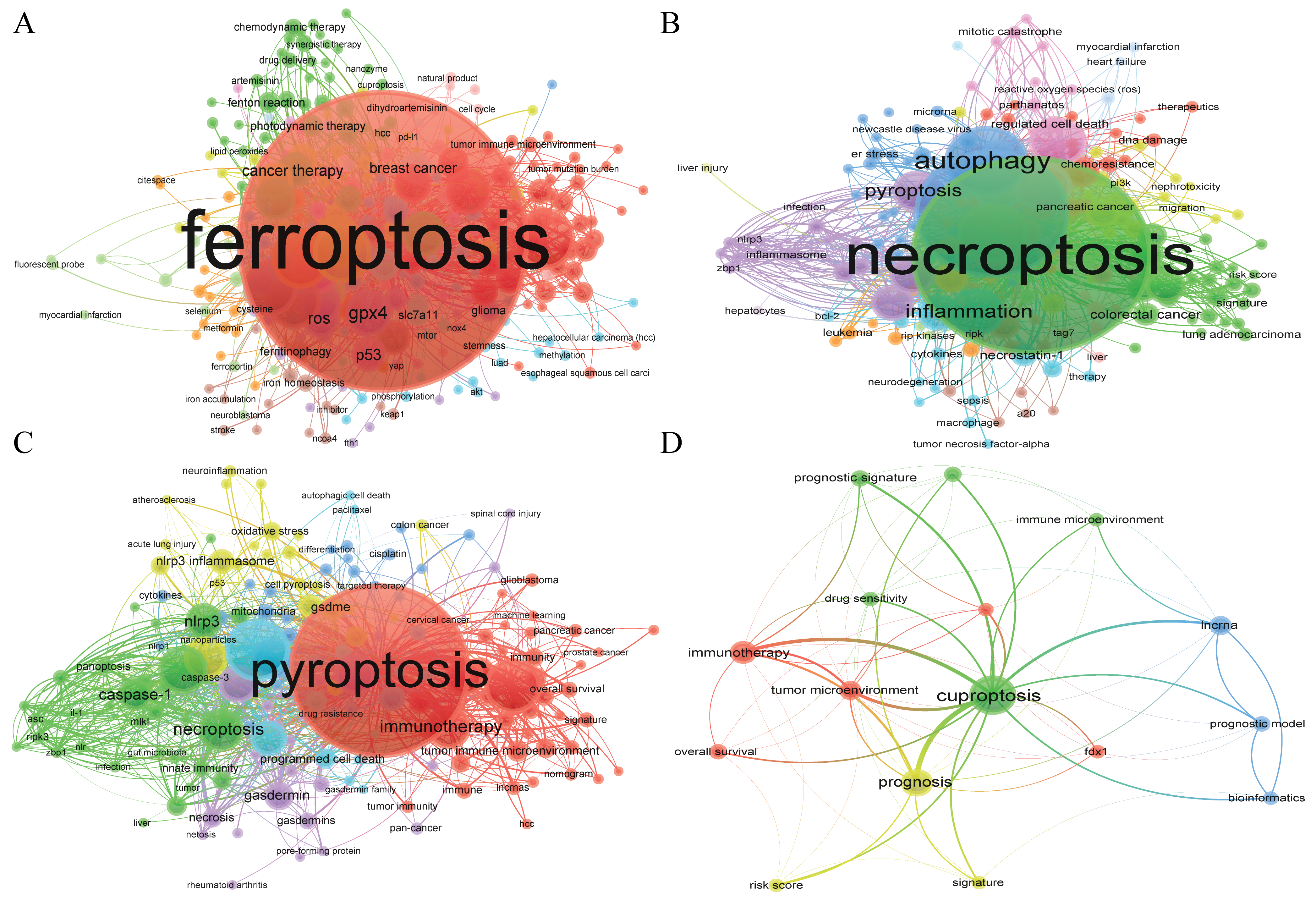

Supplement: Supplementary file 12 — Figure S10 [file 41420_2023_1421_MOESM12_ESM.tif]
